# Demands on Perceptual and Mnemonic Fidelity Are a Key Determinant of Age-Related Cognitive Decline Throughout the Lifespan

**DOI:** 10.1037/xge0001476

**Published:** 2024-01

**Authors:** Helena M. Gellersen, Jessica McMaster, Ayat Abdurahman, Jon S. Simons

**Affiliations:** 1 Department of Psychology, University of Cambridge; 2 German Center for Neurodegenerative Diseases, Magdeburg, Germany

**Keywords:** long-term memory, short-term memory, perception, aging, lifespan

## Abstract

Aging results in less detailed memories, reflecting reduced fidelity of remembered compared to real-world representations. We tested whether poorer representational fidelity across perception, short-term memory (STM), and long-term memory (LTM) are among the earliest signs of cognitive aging. Our paradigm probed target–lure object mnemonic discrimination and precision of object-location binding. Across the lifespan, cognitive deficits were observed in midlife when detailed stimulus representations were required for perceptual and short/long-term forced choice mnemonic discrimination. A continuous metric of object-location source memory combined with computational modeling demonstrated that errors in STM and LTM in middle-aged adults were largely driven by a loss of precision for retrieved memories, not necessarily by forgetting. On a trial-by-trial basis, fidelity of item and spatial information was more tightly bound in LTM compared to STM with this association being unaffected by age. Standard neuropsychological tests without demands on memory quality (digit span, verbal learning) were less sensitive to age effects than STM and LTM precision. Perceptual discrimination predicted mnemonic discrimination. Neuropsychological proxies for prefrontal executive functions correlated with STM, but not LTM fidelity. Conversely, neuropsychological indicators of hippocampal integrity correlated with mnemonic discrimination and precision of both STM and LTM, suggesting partially dissociable mechanisms of interindividual variability in STM and LTM fidelity. These findings suggest that reduced representational fidelity is a hallmark of cognitive aging across perception, STM, and LTM and can be observed from midlife onward. Continuous memory precision tasks may be promising for the early detection of subtle age-related cognitive decline.

## Public Significance Statement

Memories can vary widely in terms of fidelity, that is how accurately they represent previous experiences. Using measures of memory fidelity, this study demonstrates that a key determinant of whether middle-aged and older adults show memory deficits is whether a task requires memories to be highly detailed. Across the domains of perception, short-term memory, and long-term memory, middle-aged and older adults show poorer performance whenever they need to keep in mind highly detailed information, but largely maintain the ability to form less fine-grained representations. Tasks taxing the fidelity of perceptual and memory representations are therefore particularly sensitive to subtle changes in cognition in aging, providing a potential avenue for their use as tools for early detection of cognitive decline.

With increasing age, memory processes undergo changes that result in poorer short- and long-term retention of information (Brockmole & Logie, 2013; Cansino et al., 2018; Grady, 2012; Hedden & Gabrieli, 2004; Kensinger & Schacter, 1999; Korkki et al., 2020; Koutstaal & Schacter, 1997; Koutstaal et al., 1999; Naveh-Benjamin, 2000; Nyberg et al., 2012; Salthouse & Babcock, 1991; Simons et al., 2004; Trelle et al., 2017). However, these detrimental effects do not pertain to all aspects of memory uniformly. Age deficits are most common when a task relies on the recall of detailed, specific, multimodal, and complex stimuli and associations (Fraundorf et al., 2019; Korkki et al., 2020; Nilakantan et al., 2018; Old & Naveh-Benjamin, 2008; Perlmutter, 1979; Rhodes et al., 2020; Silver et al., 2012; Stark et al., 2019). Successful performance in these types of tasks relies on how accurately neural/cognitive processes represent previous experiences. A high-fidelity representation closely matches the detail and complexity of the experience it is intended to capture. It has been suggested that the described age deficits arise in part from a decline in forming such high-fidelity, distinct representations and binding together stimulus features into qualitatively rich memory traces that allow for the veridical reinstatement of previously experienced events (Chen & Naveh-Benjamin, 2012; Fandakova et al., 2013, 2018; Koen & Rugg, 2019; Koen et al., 2020; Korkki et al., 2022; Naveh-Benjamin & Mayr, 2018; Park et al., 2012; Trelle et al., 2017).

The idea of memory quality and specificity being among key factors underpinning episodic memory decline in old age is gaining increasing attention (Bowman et al., 2019; Fandakova et al., 2018; Giovanello & Schacter, 2012; Greene & Naveh-Benjamin, 2020; Koen & Rugg, 2019; Korkki et al., 2020; Nilakantan et al., 2018; Park et al., 2010; Rhodes et al., 2020; Sander et al., 2021; Stark et al., 2019; Trelle et al., 2019). Moreover, old age does not only result in reduced fidelity of mnemonic representations but also in a loss of the quality of representations that support perceptual processing (Gellersen, Trelle, et al., 2021; Ryan et al., 2012; Trelle et al., 2017). It is an intriguing question then whether subtle changes in the fidelity of perceptual and mnemonic representations may be among the first signs of memory decline during the cognitive aging process. To answer this question, it is necessary to include a relatively understudied phase of development: the pivotal period of midlife. It is also crucial to contrast measures of perceptual and memory fidelity with tasks that can be performed based on less precise representations. Demands on complex stimulus representations are often lacking in large-scale studies that almost exclusively utilize standard neuropsychological tests prone to ceiling effects in cognitively healthy adults (Habib et al., 2007; Nyberg et al., 2020; Salthouse, 2017). This may explain the high degree of variability in findings on cognition in midlife across the literature. There is some evidence to suggest that the degree of long-term memory (LTM) deficits in midlife may be a function of perceptual and semantic interference due to feature overlap (Güsten et al., 2021; Stark et al., 2013; Williams et al., 2019, 2020), suggesting that older age leads to less detailed memories capable of resolving interference arising from similar features. However, it is unclear whether these effects extend to the perceptual domain.

With respect to short-term memory (STM) precision, prior research has shown mixed results, with some reporting that middle-aged adults recreate features of items held in working memory less precisely (Mitchell & Cusack, 2018; Peich et al., 2013; Pertzov et al., 2015), while others find no difference (Čepukaitytė et al., 2023). It is therefore unclear if age-related decline in LTM precision may be attributable to representational changes occurring at the level of working memory. A limitation of prior studies on memory fidelity was their restricted focus on a single cognitive domain and lack of a full neuropsychological characterization of their samples based on standard cognitive tasks (besides basic dementia screening tools) to determine whether memory decline in midlife is specifically driven by demands on detailed, qualitatively rich memory representations or merely reflect general negative effects of early aging on cognition. This scarcity of research leaves our understanding of the role of a potential loss of memory fidelity in midlife incomplete.

To overcome these limitations, we probe representational quality across the domains of perception, working memory, and LTM in a lifespan sample, which allows us to determine whether a hallmark of early cognitive aging is a decline of representational fidelity regardless of the processes operating on the respective representation (for an example restricted to older adults, see Korkki et al., 2020). We employ these strategies to provide a comprehensive characterization of perceptual and memory fidelity throughout the lifespan using multiple metrics of representational quality for item-level and spatial information, hypothesizing that any task with sufficient demands on the formation, encoding, and retrieval of complex visual representations will be detrimentally affected by age, even from midlife onward. We use performance on a complex perceptual discrimination task of highly similar, simultaneously presented abstract objects to provide a proxy for the quality of visual stimulus representations (Barense et al., 2007). These tasks tap into representations formed by perirhinal and entorhinal cortices (PRC, ERC). The PRC sits at the apex of the visual ventral stream and forms unique, viewpoint-invariant representations of objects to resolve feature interference between highly similar stimuli, which are then relayed to the hippocampus via the entorhinal cortex (Bussey & Saksida, 2002; Graham et al., 2010; O’Neil et al., 2015; Watson & Lee, 2013). A decline in the quality of this hippocampal input is expected to also impact the fidelity of memory encoding. It is therefore of particular interest to extend our previous investigation of changes in complex perceptual discrimination in older adults to midlife (Gellersen, Trelle, et al., 2021; Trelle et al., 2017). We also include a condition in which we control for basic visual discrimination ability under lower levels of feature similarity, predicting age-related deficits only in the high-ambiguity condition. Using this task, we test whether potential deficits in object recognition memory in midlife are associated with deficits in the ability to form high-quality representations of visual stimuli at an earlier processing stage than LTM (Gellersen et al., 2023; Gellersen, Trelle, et al., 2021).

For our two measures of memory fidelity, we designed a novel task that combines aspects of object mnemonic discrimination and precision of relational binding for object locations in a scene (Gellersen, Coughlan, et al., 2021). We employ both an STM and a LTM version of the same paradigm to determine whether study-test delay might drive the magnitude of age-related memory fidelity deficits. This task includes object recognition memory trials using a mnemonic similarity task format in which highly similar lures and targets are to be distinguished. Mnemonic discrimination has been proposed as a behavioral measure of pattern separation, the ability of the medial temporal lobe (MTL) and the hippocampus, in particular, to encode and retrieve distinctive memory representations (Kirwan & Stark, 2007; Stark et al., 2019). The mnemonic discrimination metric is well suited to test for detailed object representations and for vulnerability to interference from feature overlap from other stimuli (Reagh & Yassa, 2014; Stark et al., 2019; Trelle et al., 2017). We chose to present target and lures simultaneously as to reduce the contribution of prefrontal cortex (PFC)-dependent processes (Gellersen, 2023; Gellersen, Trelle, et al., 2021) while maintaining demands on high-fidelity stimulus representations.

We also probe the STM and LTM fidelity of spatial information associated with the same objects using an analog report paradigm for source memory in which participants recreate object positions as precisely as possible (Bays & Husain, 2008; Richter et al., 2016). This task overcomes the limitation of many memory tests that only differentiate between successful and unsuccessful retrieval and thus do not reflect the wide range over which the fidelity of memory may vary within and between individuals (Bays et al., 2009; Brady et al., 2013; Harlow & Donaldson, 2013; Korkki et al., 2020; Nilakantan et al., 2018; Peich et al., 2013; Pertzov et al., 2015; Richter et al., 2016; Zhang & Luck, 2008). Memory precision tasks provide a continuous memory error metric by providing an exact measure of the distance between the target feature of an object and its real-life counterpart. Recent evidence employing these types of tasks has found no STM or LTM object-location memory deficit in middle-aged adults when assessing performance based on mean target-response distance (Čepukaitytė et al., 2023). However, a metric of absolute mean target-response distance includes information from both remembered and forgotten trials meaning that coarse-grained memory representations may not be distinguished from guessing. The measure can therefore not provide insights into the sources of memory errors. We use a behavioral mixture modeling approach that has the advantage of distinguishing trials in which participants could successfully retrieve spatial information from those in which they are likely to have guessed. As a result, this method allows us to determine whether age-related memory deficits are due to increased forgetting and/or a decline in the fidelity with which mnemonic representations are retrieved (Bays & Husain, 2008; Richter et al., 2016). Based on prior findings in older adults (Korkki et al., 2020; Nilakantan et al., 2018), we hypothesized that this modeling approach would be more sensitive to subtle memory fidelity declines in midlife, showing that age deficits in this group may not be due to forgetting but diminished mnemonic precision.

Because our paradigm uses stimulus displays with trial-unique everyday objects rather than minimalistic shape stimuli typically used in working memory analog tasks (Manga et al., 2021; Pertzov et al., 2015; Zokaei, Čepukaitytė, et al., 2019), it closely matches encoding demands and perceptual features across short and long delays (see also Korkki et al., 2020; Lugtmeijer et al., 2019; Rhodes et al., 2020), allowing us to compare memory performance in STM and LTM versions of the task. Moreover, memory processes underpinning the recall of object and object-location information, respectively, are only partially overlapping (Clark et al., 2017; Cooper & Ritchey, 2019; Stark et al., 2019; Stevenson et al., 2020; Wais et al., 2018) and are differently affected by age (Bouffard et al., 2023; Tran et al., 2021). Therefore, incorporating measures for the fidelity of object and object-location recall allows for a comparison of age effects on the precision of both types of representations and makes it possible to test for an association between fidelity of item recognition and source memory from the same encoding period (Kim & Yassa, 2013; Richter, 2020). Although prior studies have included similar metrics, they either did not include precision estimates (Kim & Yassa, 2013) or focused solely on younger adults (Richter, 2020). It is therefore unclear whether the trial-by-trial association between fidelity of object and spatial memory follows the same patterns as that observed for course-grained mnemonic representations. Given prior work suggesting that stimulus features in working memory may be forgotten independently from one another (Bays et al., 2011; Fougnie & Alvarez, 2011; Markov et al., 2021), whereas pattern completion and reinstatement tend to result in holistic retrieval from LTM (Grande et al., 2019; Horner et al., 2015), we predicted a stronger association between fidelity of object and spatial information in LTM as compared to STM. Moreover, based on well-documented age-related declines in binding together elements in memory (Chen & Naveh-Benjamin, 2012; Henkel et al., 1998; Hou et al., 2019; James et al., 2019; Lyle et al., 2006; Naveh-Benjamin & Mayr, 2018; Ngo & Newcombe, 2021), we also hypothesized a weaker trial-by-trial association between object and spatial memory fidelity with age.

Finally, as previously touched upon, most prior studies of precision memory in cognitive aging did not include a detailed battery of standardized neuropsychological tasks across multiple functional domains (Mitchell & Cusack, 2018; Nilakantan et al., 2018; Peich et al., 2013; Pertzov et al., 2015). Claims that demands on high-fidelity stimulus representations constitute a decisive and specific factor in driving age-related cognitive deficits could further be strengthened if it can also be demonstrated in the same participants that standard neuropsychological tasks and tests that allow for reliance on coarse-grained representations are not as sensitive to age-related declines. We therefore provide a neuropsychological characterization of our lifespan sample, presenting a comprehensive overview of the magnitude of age effects across cognitive domains. We further identify neuropsychological correlates of individual differences in memory fidelity, focusing on measures of MTL and PFC integrity. Performance on standard memory tasks known to index hippocampal integrity may contribute to explaining interindividual differences in memory quality and particularly the ability to retrieve high-fidelity memories after longer delays (Andersson et al., 2006; Schmidt, 1996; Shin et al., 2006; Tabatabaei-Jafari et al., 2020). Prefrontally-mediated executive functions may also contribute to age-related memory fidelity declines, given that PFC is highly involved in strategic memory encoding and retrieval (Cohn et al., 2008; Shing et al., 2008; Trelle et al., 2017), counteracts false memories for highly similar information (Devitt & Schacter, 2016; Fandakova et al., 2018; Foster et al., 2020; Trelle et al., 2017), and is essential for source memory (Dobbins et al., 2002; Duarte et al., 2005; Henkel et al., 1998). These contributions are likely particularly important for the maintenance of high-fidelity working memory representations. A combination of these neuropsychological measures may therefore account for interindividual variability in, and age effects on, mnemonic fidelity.

## Summary of the Aims and Hypotheses of the Current Study

We provide a comprehensive assessment of representational and mnemonic fidelity by spanning our investigation across cognitive domains in a lifespan sample to determine whether a loss of representational fidelity with age is ubiquitous and begins to emerge in midlife. First, we determine whether age-related deficits can be found across all tasks with demands on detailed, complex representations and whether memory fidelity metrics can uncover subtle age effects not discernible when using standard neuropsychological assessments. Specifically, according to our primary hypothesis, we predict poorer performance in middle-aged and older adults for the perceptual and mnemonic discrimination of highly similar stimuli and for the precision but not success of recall of object-location information. In line with this hypothesis, we predict that a precision metric derived from mixture modeling provides the most sensitive memory quality index by accounting for memory errors due to forgetting. Second, capitalizing on our new experimental paradigm, we aim to examine the trial-by-trial relationship between item and contextual memory fidelity. A secondary hypothesis therefore predicts this association to be decreased with age and the coupling between these two types of memory content to be stronger in LTM as opposed to STM. Finally, we provide a neuropsychological characterization of individual differences in memory fidelity across the lifespan, expecting executive functions (proxies of PFC) and delayed memory scores (proxy for hippocampal functions) to serve as predictors of STM and LTM fidelity, respectively.

## Method

### Participants

We recruited 132 volunteers between the ages of 18 and 85. Participants consisted of 30 young participants (18–35 years old), 50 middle-aged adults (36–59 years), and 52 older adults (60+ years). To increase the representativeness of our sample, recruitment was carried out through multiple channels: all universities in the city of Cambridge, United Kingdom, churches, social clubs, community centers, notice boards of shops, handing out flyers in popular areas of the city, and using Facebook advertisements for the greater Cambridge area and nearby towns. Participants were native English speakers with normal or corrected-to-normal vision, no color blindness, no developmental conditions, and no current diagnosis of psychiatric or neurological conditions. Participants were excluded if they received neuropsychological test scores that put them at risk for developing mild cognitive impairment. Age groups did not differ in terms of education, *F*(2, 129) = 1.09, *p* = .340, but middle-aged and older groups included more female participants (which was accounted for in sensitivity analyses). The study was approved by the Cambridge Psychology Research Ethics Committee and complies with the APA ethical standards in the treatment of participants. Sample demographics can be found in Table 1.[Table tbl1]

The present sample size allows us to detect age group effect sizes of partial η_*p*_^2^ = .25 (as reported by Korkki et al., 2020) in a mixed ANOVA with .95 power assuming α = .05 (according to G*Power Version 3.1; Faul et al., 2009).

In addition to the data presented here, we also collected information on lifestyle from the same participants, which will be analyzed in a separate project as these measures are beyond the scope of this project.

### Precision Memory Task

We designed a new paradigm to assess different aspects of memory fidelity, details of which have previously been described in Gellersen, Coughlan, et al. (2021). We refer to this paradigm as the “precision memory task.” The task requires encoding the location and identity of multiple objects on a display and tests discrimination of studied objects from highly similar lures at retrieval, followed by a visuospatial reconstruction test in which object locations are to be recreated as precisely as possible. The task therefore contains two different trial types that provide indices of context-free object recognition memory and associative/source memory, respectively. Object recognition memory is here described as “mnemonic discrimination” given that the trials involve target–lure discrimination following the well-established mnemonic similarity task (Stark et al., 2019). Performance on object-location binding trials is indexed with three different measures throughout this study: “retrieval success” and “precision” derived from mixture modeling (see below for details), which describe the likelihood of memory retrieval and its fidelity, respectively, and “mean absolute localization error” (short “mean error” or “localization error”), which is a model-free metric describing the target-response distance.

Note that we decided to present the mnemonic discrimination question in a forced choice rather than a yes/no format to reduce demands on PFC-dependent strategic retrieval processes and ensure a relatively greater contribution of perirhinal–entorhinal processes underpinning representational quality of complex objects (O’Neil et al., 2015; Watson & Lee, 2013).

#### Materials

Stimuli for the precision memory tasks consisted of 150 pairs of everyday objects, 150 single objects, and 100 background images obtained from either the Konklab image repository (https://konklab.fas.harvard.edu) or through Google Image Search (Mountain View, California, United States). Seventy-five of the background images, 75 of the object pairs, and all 150 single objects were allocated to the STM precision task, while the remaining 25 backgrounds and 75 object pairs belonged to the LTM version. This allocation was required to ensure 75 test objects for each task format where one item per display was tested for the short-term version and all three items per display were tested in the long-term version.

Each object pair consisted of two different exemplars of the same kind of object (e.g., book). For each pair, one was randomly determined to be the “target,” while the other was chosen to be a “lure” item. Each pair was rated by an independent cohort (see Section 1 and Figure S1 in the online supplemental materials) to ensure that items were distinguishable, yet similar. Based on these ratings, object pairs were randomly allocated to the short- or long-term task while ensuring that overall target–lure similarity was matched between the two task formats. Only the target objects were presented on the displays. Backgrounds were chosen to display uniform patterns. Three target objects were randomly combined with one background image to create a stimulus display. Objects were placed in pseudo-random locations on an invisible circle centered in the middle of the background image. The randomization procedure was constrained to ensure no bias in the positions of objects to avoid systematically influencing responses. Separation between objects was a minimum of 62.04° to ensure that objects would not overlap. Displays were identical for all participants but the order of presentation at the study and test, respectively, was randomized across subjects.

#### Procedure

**Figure 1 fig1:**
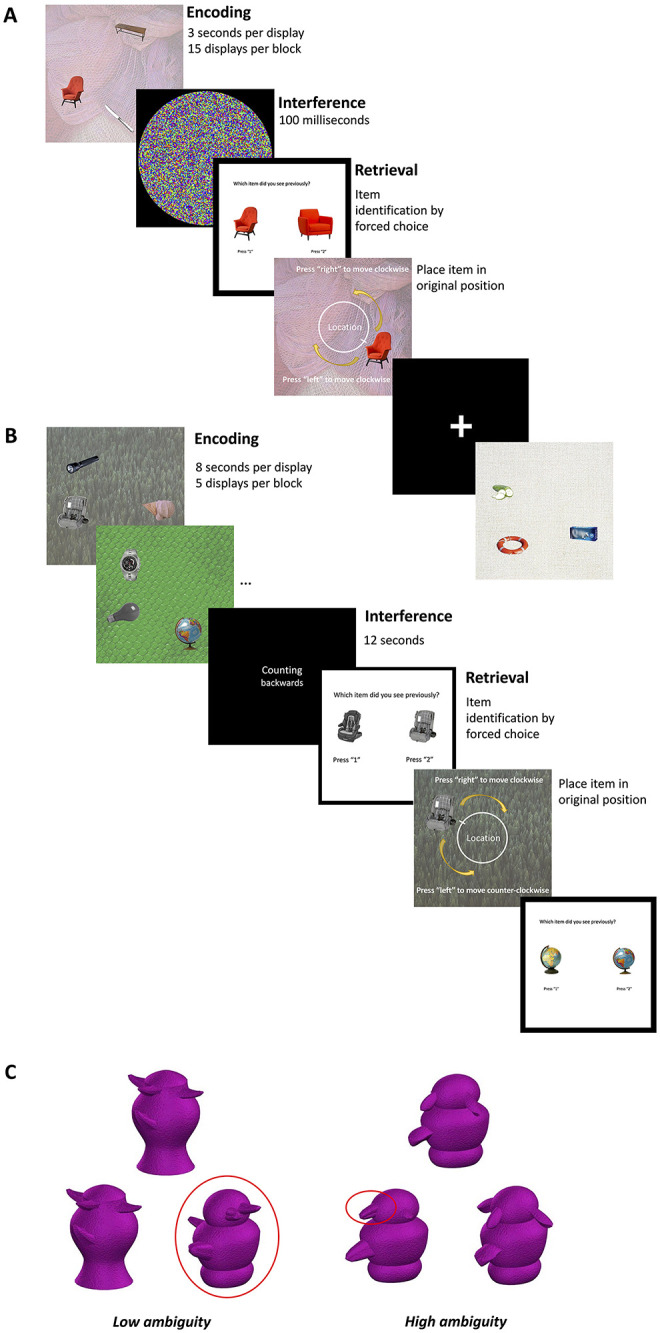
Schematic of Memory and Perception Tasks *Note*. (A) Short-term precision memory task. (B) Long-term precision memory task (adapted from “Memory Precision of Object-Location Binding Is Unimpaired in APOE ε4-Carriers With Spatial Navigation Deficits,” by H. M. Gellersen, G. Coughlan, M. Hornberger, and J. S. Simons, 2021, *Brain Communications*, *3*(2), Article fcab087 (https://doi.org/10.1093/braincomms/fcab087). Copyright 2021 by the Oxford University Press on behalf of the Guarantors of Brain). Stimuli for these tasks were obtained from https://konklab.fas.harvard.edu/# and are printed with permission. Copyright Professor Talia Konkle. (C) Perceptual discrimination of objects, showing differences between candidate novel objects within the red circle (not shown to participants; adapted from “The Human Medial Temporal Lobe Processes Online Representations of Complex Objects,” by M. D. Barense, D. Gaffan, and K. S. Graham, 2007, *Neuropsychologia*, *45*(13), pp. 2963–2974 (https://doi.org/10.1016/j.neuropsychologia.2007.05.023). Copyright 2007 by Elsevier; “Executive Function and High Ambiguity Perceptual Discrimination Contribute to Individual Differences in Mnemonic Discrimination in Older Adults,” by H. M. Gellersen, A. N. Trelle, R. N. Henson, and J. S. Simons, 2021, *Cognition*, *209*, Article 104556 (https://doi.org/10.1016/j.cognition.2020.104556). Copyright 2021 by the Elsevier B.V.). Individual Greeble stimuli originate from https://www.tarrlab.org/ and are printed with permission. Copyright Professor Michael J. Tarr. See the online article for the color version of this figure.

Both memory tasks began with a practice phase during which the experimenter emphasized the importance of stimulus detail (Figure 1A and B). Participants were told to aim at recreating the original position of the test objects as precisely as possible during the location task. After successful completion of the practice phase, participants moved on to the main task. The order in which participants completed the STM and LTM tasks was counterbalanced. In the short-term task, participants saw 15 displays in each of the five blocks adding up to a total of 75 trials. A display consisted of three objects on a background with a uniform pattern. Only one of the items on that display was tested in each STM trial. After a given display was presented for 3 s, a visual mask appeared for 100 ms. Then, the test phase began with the two-alternative forced choice object mnemonic discrimination question where the target object was presented next to the corresponding lure item on a white background. Participants pressed the “1” key to endorse the item on the left-hand side of the screen as old and the “2” key to choose the right-hand item. Regardless of whether participants chose the correct item, they moved on to the location precision question. The item chosen in the identification question was carried over to the localization question, even if participants incorrectly identified the lure item. This was done to avoid any kind of feedback regarding memory performance throughout the task. The object appeared on the corresponding encoding display in a random location on a white dial centered around the midpoint of the display. In the middle of the dial, the word “Location” printed in white cued participants to the objective of the task. Participants used the arrow keys to move the object clockwise (arrow pointing to the right) or counterclockwise (arrow to the left) around the dial. There was no time limit, but participants were encouraged to respond within 15 s before the location cue turned red to keep response times relatively comparable between participants. Participants logged their response by pressing the space bar.[Fig fig1]

The long-term precision memory task consisted of five blocks each including a study and test phase. During the study, participants viewed five displays in a row for 8 s each. Encoding displays were separated by a fixation cross, which appeared for 1 s. The study phase was followed by an interference task where participants were asked to count backward in multiples of three from a random number between 50 and 100 for 12 s to prevent rehearsal of memory content before the test phase. Participants then completed 15 test trials, which followed the same procedure as the analogous STM precision task. For each of the encoding displays, all three objects were tested in sequence using the same procedure and response options as in the short-term precision task. The precision memory tasks were run on Psychtoolbox-3 (http://psychtoolbox.org; Kleiner et al., 2007) on MATLAB (Mathworks, Inc., United States). Test order for STM and LTM was randomized across participants.

For mnemonic discrimination, we computed *d*′ with the dprime.mAFC function in the R package psyphy (Knoblauch, 2021).

### Object Perceptual Discrimination Task

The details for the object discrimination task have been described previously (Barense et al., 2007; Gellersen, Trelle, et al., 2021). Briefly, the task required the discrimination of three abstract object (greebles) stimuli per trial, either under conditions of low or high feature overlap. Only the high-ambiguity task has been shown to require the perirhinal cortex to disambiguate similar complex feature conjunctions (Barense et al., 2007). The low-ambiguity condition was used as a control to exclude individuals with poor performance that may be driven by impaired basic visual processes. Perceptual discrimination tasks were completed after the precision memory tests. The task consisted of 10 practice trials and 60 test trials with 36 belonging to the high and 24 to the low-ambiguity condition. Participants were told to identify the odd one out of the three exemplars, stressing that two greebles were always identical but slightly rotated. During the training phase, the experimenter pointed to the differences between exemplars after participants provided an answer to ensure a clear understanding of the task. We computed *d*′ with the dprime.oddity function in the R package psyphy (Knoblauch, 2021). Three older adults performed at chance level in the high-ambiguity task despite >87% accuracy in the low-ambiguity task. Given that *d*′ oddity calculation fails for below chance performance, their responses were recoded as 1 − 1/(2 × number of trials; Macmillan & Creelman, 1991).

The perceptual discrimination task was implemented using COGENT (2000) for MATLAB (Mathworks, Inc., United States). Greeble stimuli are available at https://sites.google.com/andrew.cmu.edu/tarrlab/stimuli?authuser=0. 

### Neuropsychological Tests

After the computer-based memory and perception tasks, participants completed a standardized neuropsychological test battery comprised of the Rey–Osterrieth complex figure test (ROCFT; Osterrieth, 1944; Rey, 1941), the Rey auditory verbal learning test (RAVLT), the Addenbrooke’s cognitive examination (ACE; Mioshi et al., 2006), the trail making tests A and B (Delis et al., 2001), and the digit span forward and backward (Wechsler, 2008). The digit span tests, the trails tests, and the number of words produced in the verbal fluency test of the ACE were used to derive a composite executive functioning test that was expressed in terms of *Z*-scores calculated across the full sample in accordance with prior work (Gellersen, Trelle, et al., 2021; Trelle et al., 2017). The three performance metrics were moderately correlated (.27 = <|*r*| = <.33; all *p* < .01). We also computed a composite normalized delayed memory score from ROCFT and RAVLT performance.

### Mixture Modeling: Which Processes Best Capture Memory Performance?

**Figure 2 fig2:**
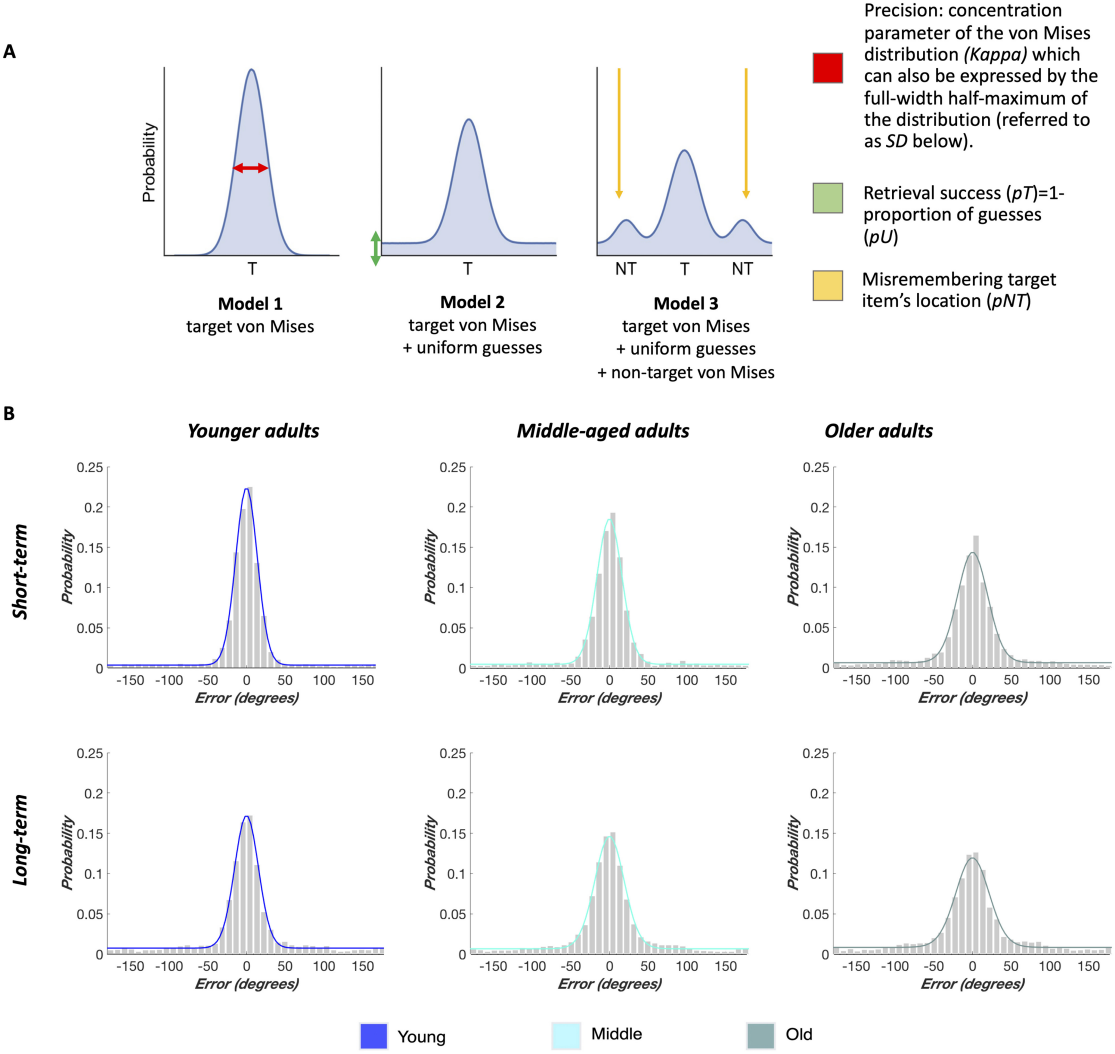
Tested Models and Results From the Mixture Modeling Approach *Note*. (A) Proposed models to capture location memory performance. (B) Standard mixture models best fit localization error responses, which are here shown separately for short- and long-term memory tasks from modeling across all participants in a given age group. Final chosen model parameters correspond to the respective maximum a posteriori values derived from Bayesian mixture modeling (see Section 2 in the online supplemental materials for details). See the online article for the color version of this figure.

Responses in the localization task may reflect different retrieval mechanisms in a given trial: (a) recall of object locations for which the underlying representations vary from fine- to coarse-grained (i.e., high to low precision), (b) random guesses, or (c) misbinding errors in which the location of the target item is confused with that of another object also presented on the same display. When the target-response errors can be fitted with a model that includes misbinding errors, it suggests that a person has access to location information (with varying degrees of precision) that they were not able to bind to the correct item. It is, therefore, crucial to account for this possibility given that large mean localization errors on their own may look as though recall of spatial information failed entirely rather than spatial information being stored independently of its original item. We fit probabilistic mixture models to the location placement errors expressed as the degrees separating the response from the target to determine which retrieval mechanisms best describe the distribution of trial responses (see Figure 2A). We used Bayesian mixture modeling implemented with the MemToolbox in MATLAB 2016a for model estimation and selection (Suchow et al., 2013). Following the model selection procedure detailed in Section 2 in the online supplemental materials, we chose the standard mixture model with estimates of retrieval success and precision to describe responses in the two memory tasks. The proportion of trials within the uniform distribution represents the guess rate *pU* and 1 − *pU* therefore expressing retrieval success *pT*. A larger full-width half-maximum (*SD*) of the von Mises distribution (circular Gaussian around the location parameter space) corresponds to a lower fidelity of the recalled responses. The MemToolbox modeling procedure returns the guess rate and *SD* such that higher values represent poorer performance. To facilitate the interpretation of our analyses on single-subject data, we used *pT* instead of the guess rate and converted the imprecision *SD* metric into the concentration parameter kappa (κ) representing memory precision. The *SD* to κ conversion was achieved using the *sd2k* function from https://www.paulbays.com/toolbox/. In both cases, higher values indicate better performance.[Fig fig2]

### Statistical Analyses

Data were analyzed using R Studio, Version 4.2.1 (R Core Team, 2022). We used the following R packages for data wrangling, analysis, and visualization: dplyr (Wickham et al., 2023), plyr (Wickham, 2011), ggplot2 (Wickham, 2016), ggcorrplot (Kassambara, 2019), ggpubr (Kassambara, 2020), raincloudplots (Allen et al., 2021), sjPlot (Lüdecke, 2022), ez (Lawrence, 2016), afex (Singmann et al., 2022), car (Fox & Weisberg, 2019), lme4 (Bates et al., 2015), effects (Fox & Weisberg, 2019), effectsize (Ben-Shachar et al., 2020), emmeans (Lenth, 2022), psych (Revelle, 2022), rstatix (Kassambara, 2021), MASS (Venables & Ripley, 2002), purrr (Wickham & Henry, 2023), bootES (Gerlanc & Kirby, 2021), pastecs (Grosjean & Ibanez, 2018), expss (Demin, 2022), performance (Lüdecke et al., 2021), table1 (Rich, 2021), and formattable (Ren & Russell, 2021). This study was not preregistered.

We excluded two middle-aged adults from the analysis with model-derived estimates for LTM due to poor model fit or because low retrieval success artificially inflated the κ parameter due to an insufficient number of trials within the von Mises distribution (leaving a total of 128 participants with usable data for this task after another two participants were excluded because of technical failures). We also excluded three older adults from the analysis on the STM task for the same reasons (leaving a total of 129 participants with usable data for this task). One older adult was excluded from analyses including the executive functioning composite because they paused during the Trail B task to comment on the time pressure therefore leading to an inflated time to complete the trail (*z* = −6.87; leaving a total of 131 participants with usable data for this task).

#### Age Effects on Mnemonic and Perceptual Object Discrimination

For object identification in the mnemonic discrimination tasks, we probed the effects of age group and study-test delay (short, long) in a mixed ANOVA. Age group effects on perceptual discrimination of objects were assessed using a one-way between-subjects ANOVA. Post hoc comparisons were adjusted using the Tukey method.

#### Models for Age Effects on Object-Location Memory

We determined whether age-dependent declines in memory precision could be observed at the subject level and whether age differences in the precision of object-location binding outweigh those in retrieval success, using a mixed ANOVA on object localization performance with age group (young, middle, old) as a between-subjects factor and delay (STM, LTM) and parameter (retrieval success, precision) as within-subjects factors. To provide further support for our hypothesis that a precision metric obtained via mixture modeling is more sensitive than mean localization error, we ran a mixed ANOVA with age group as a between-subjects factor and method (mixture modeling, no modeling) and delay (short, long) as within-subjects factors on scaled precision and mean absolute localization error data.

#### Mixed Linear Models for the Association Between Object and Object-Location Memory Fidelity

Next, we determined to what extent the fidelity of intraobject information is associated with the fidelity of object-location binding on a trial-by-trial basis and whether the study-test delay impacts the degree to which the fidelity of item-based and spatial representations are linked in memory. A mixed linear model was fit on target-response error data for individual trials with age group as between-subjects fixed factor, delay (short, long) as within-subjects factor, object identification accuracy as continuous between-subjects fixed factor, and trial number and participants as random effect.

#### A Neuropsychological Characterization of Retrieval Success and Precision

We conducted a neuropsychological characterization of our sample to test our hypothesis that memory fidelity measures could uncover subtle age differences not visible with standard test scores. We further aimed to identify which factors explain individual variability in mnemonic discrimination and memory precision in STM and LTM. We focused on proxies of prefrontal and MTL function as measured by the executive functioning and the memory composite scores (see above), respectively. We also aimed to reproduce our previous finding that complex object perception is a predictor of age-related mnemonic discrimination deficits in older adults (Gellersen, Trelle, et al., 2021) and to extend this to the middle-aged group. We ran separate models on mnemonic discrimination *d*′ and localization as measured by *pT* and κ. All models were controlled for age and years of education.

### Openness and Transparency

In accordance with the Transparency and Openness Promotion Guidelines, all data, software code, and other methods developed by others are appropriately acknowledged. Materials pertaining to this study are available on the Open Science Framework at https://osf.io/24vqk/ (Gellersen, 2023; data and analysis code), except for the stimuli for the precision task (which are available on https://konklab.fas.harvard.edu/#) and for the perceptual task (which were kindly provided to the Cambridge Memory Lab by Morgan Barense and can be found at https://sites.google.com/andrew.cmu.edu/tarrlab/stimuli?authuser=0). Code for the memory and perception tasks are available upon request. The hypothesis and analysis plan were not preregistered but we clearly state which analyses were hypothesis-driven (i.e., preplanned) and which were exploratory.

## Results

**Figure 3 fig3:**
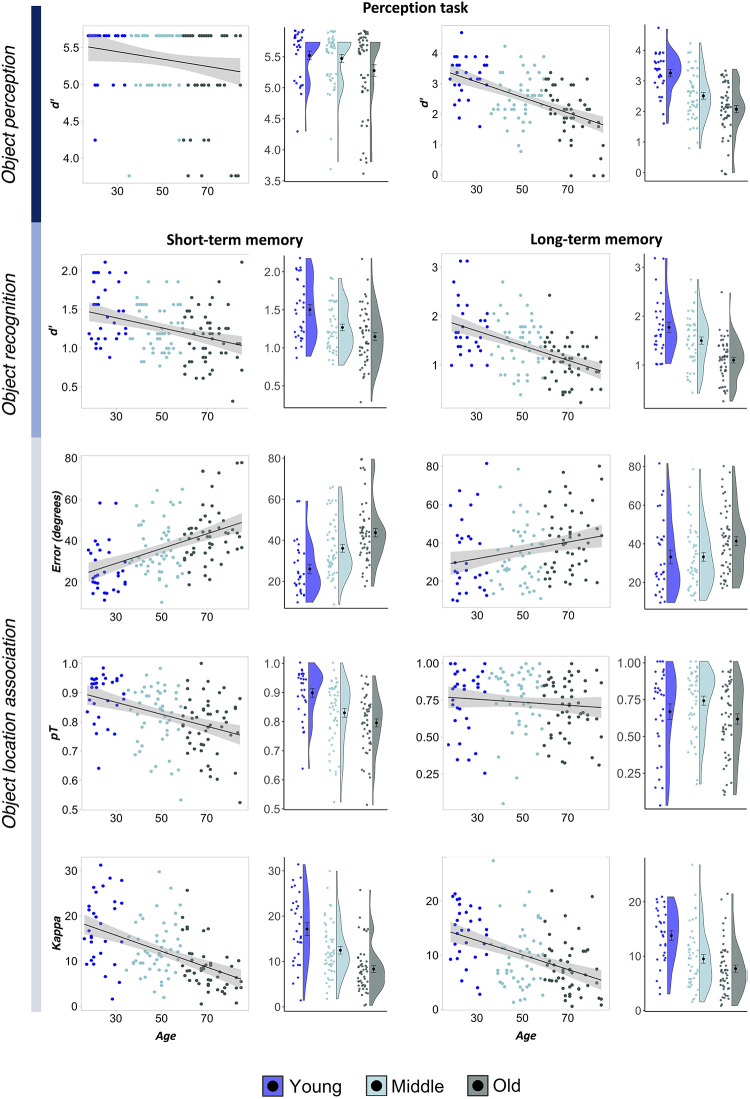
Summary of Continuous and Group-Based Effects of Age on Performance in Perceptual Discrimination (d′), Mnemonic Discrimination (d′), and Object-Location Memory (Retrieval Success pT, Precision κ, Mean Absolute Error) *Note*. Memory scores are split up by task (short-term vs. long-term memory). Scatter plots show linear trend lines with standard error of the mean. Raincloud plots show individual data points and their distribution alongside mean and standard error for cognitive performance metrics in each age group. Object perception as indexed using the perceptual discrimination task is scored using *d*′ for an oddity task with three exemplars. Object recognition as indexed by mnemonic discrimination tasks is scored using *d*′ for two-alternative forced choice response options. Mean absolute error is expressed in degrees between the target location and the response given by participants. *pT* refers to retrieval success, that is, the proportion of trials (%) in which participants were likely to retrieve object-location information. Kappa refers to the precision with which object locations were reproduced only in those trials in which participants did not guess. For better visualization, one extreme outlier in the older adult group (error ∼80°) was removed from the plot for short-term memory mean error. See the online article for the color version of this figure.

Summary statistics for performance on memory and discrimination performance by age group can be found in Table 2. Figure 3 provides an overview of age-related differences in performance across perceptual and mnemonic fidelity metrics.[Table tbl2][Fig fig3]

### Do Changes in Complex Perception and Memory Fidelity Occur in Midlife?

We expected that tasks requiring detailed, high-fidelity stimulus representations are most sensitive to early detrimental cognitive changes emerging in midlife. Specifically, we expected poorer performance in middle-aged and older adults in high-ambiguity object perceptual discrimination, object mnemonic discrimination, and the precision of object-location associative memory, but spared performance in low-ambiguity perceptual oddity tasks, standard neuropsychological tasks, and gist-based memory for object locations.

#### Perceptual and Mnemonic Object Discrimination

Age effects on perceptual discrimination are shown in the upper panel of Figure 3. All participants scored >87% accuracy (*d*′ = 3.76) in the low-ambiguity object discrimination task. Due to a highly skewed distribution of *d*′ scores in the low-ambiguity condition, we used a Kruskal–Wallis rank sum test, finding no significant age effect, χ^2^(2) = 2.51, *p* = .286. A one-way ANOVA on high-ambiguity discrimination scores found a large effect of age group, *F*(2, 125) = 21.84, *p* < .001, η_*p*_^2^ = .26, with younger adults outperforming middle-aged adults, *t*(125) = 4.17, estimate = 0.747, 95% CI [0.322, 1.172], *p* *<* *.*001, *d* = −1.06, who in turn performed better than older adults, *t*(125) = 2.82, estimate = 0.433, [0.067, 0.798], *p* = .015, *d* = −0.55.

Age effects on mnemonic discrimination are shown in the middle panel of Figure 3. A mixed ANOVA on object mnemonic discrimination *d*′ scores revealed a main effect of age group, *F*(2, 127) = 20.25, *p* < .001, η_*p*_^2^ = .24, and delay, *F*(1, 127) = 29.77, *p* < .001, η_*p*_^2^ = .19, as well as an interaction between age group and delay, *F*(2, 127) = 4.19, *p* = .017, η_*p*_^2^ = .06. Post hoc tests with the Sidak correction showed that this interaction was due to the age difference between middle-aged and older adults being larger in the LTM task, *t*(127) = 2.34, estimate = 0.223, 95% CI [0.035, 0.411], *p* = .021, *d* = 0.42, with older adults only performing worse on the long-, *t*(127) = 3.64, estimate = 0.346, [0.121, 0.571], *p* = .001, *d* = 0.65, but not the STM task compared to middle-aged adults, *t*(127) = 1.83, estimate = 0.123, [−0.037, 0.283], *p* = .165, *d* = 0.32. However, younger adults did outperform older adults on the short-term mnemonic discrimination task, *t*(127) = 4.50, estimate = 0.349, [0.165, 0.532], *p* < .001, *d* = 0.80. There was also a significant age difference between middle-aged and younger adults in mnemonic discrimination performance, STM: *t*(127) = 2.89, estimate = 0.225, [0.040, 0.411], *p* = .013, *d* = 0.51; LTM: *t*(127) = 2.55, estimate = 0.280, [0.019, 0.541], *p* = .032, *d* = 0.45, which was equivalent across tasks, *t*(127) = .50, estimate = 0.055, [−0.163, 0.272], *p* = .621, *d* = 0.09.

A follow-up analysis was conducted to address a reviewer comment which suggested that there may be an interaction between target–lure similarity and age group differences such that the highest degree of target–lure similarity would lead to poor performance in all age groups, whereas moderate similarity would show the expected age effects (Stark et al., 2013; Yassa et al., 2011). There was some support for a similarity by age effect in a trial-by-trial analysis, *F*(4, 18,623) = 3.25 *p* = .011, but due to low and high similarity bins having significantly fewer trials than the moderate similarity bin, we do not place high confidence in this result (see Section 3 and Figure S4 in the online supplemental materials).

#### Precision of Object-Location Association

Age effects on the three measures of object-location memory are shown in Figure 3. We first compared model estimates for each age group as derived from mixture modeling across all participants in a group. We computed the percent overlap for each pairwise comparison of distributions for likely model estimates derived from Bayesian modeling (Section 2.2 in the online supplemental materials; Pastore, 2018). On a group level, credible estimates for retrieval success and precision were completely nonoverlapping between all three age groups for STM. The same was true for precision, but not retrieval success, in the LTM task. These results provide substantial evidence that, in all pairwise comparisons, middle-aged and older adults exhibit a reduction in memory precision at the group level (see also Figure 2B).

For model estimates on single-subject data, a mixed ANOVA found a significant main effect of age group, *F*(2, 122) = 21.09, *p* < .001, η_*p*_^2^ = .26; but none for parameter (*pT*, κ) and delay (short, long) given that data were *z*-scored and therefore had identical means and standard deviations. The model also contained significant two-way interactions between age group and memory process, *F*(2, 122) = 4.53, *p* = .013, η_*p*_^2^ = .07, and between age group and delay, *F*(2, 122) = 4.15, *p* = .018, η_*p*_^2^ = .06, as well as significant three-way interaction of age group, delay, and parameter, *F*(2, 122) = 4.20, *p* = .017, η_*p*_^2^ = .06.

**Figure 4 fig4:**
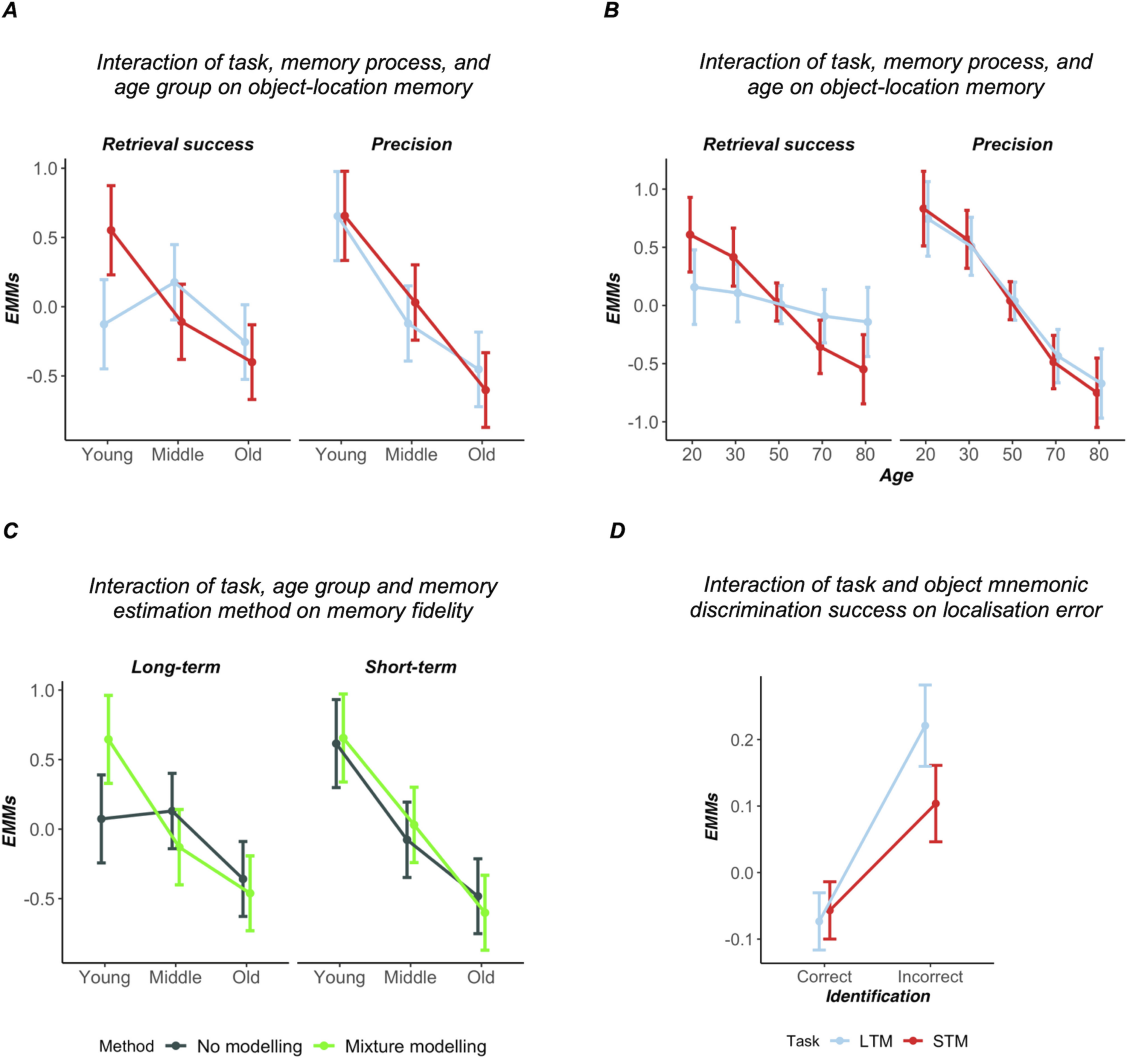
EMMs and Their 95% Confidence Intervals for Models of Interest *Note*. (A) A model comparing age group effects for performance based on mixture modeling contains a three-way interaction of memory process (*pT*, κ), age group (young, middle, old), and task (short-term, long-term memory). (B) The same model when using age as a continuous variable. (C) The model comparing different metrics used to index memory fidelity of object-location binding contains a three-way interaction of age group, task (short-term vs. long-term memory), and method (mean localization errors without mixture modeling versus estimates of κ derived from mixture modeling). (D) When examining the trial-by-trial relationship between item and spatial fidelity, a model on target-response errors contained an interaction of task (short-term, long-term memory) and object mnemonic discrimination on a given trial (correct vs. incorrect). EMMs = estimated marginal means. See the online article for the color version of this figure.

We followed up on this three-way interaction by contrasting the magnitude of age differences in precision and retrieval success between young and middle-aged and the middle-aged and older adults, respectively, and by determining whether the effect is further driven by delay. The plot of estimated marginal means for the three-way interaction is shown in Figure 4A. Memory declines in precision in middle-aged adults were greater than those in retrieval success compared to younger adults and the magnitude of this difference depended on delay, *t*(122) = 2.90, estimate = 1.11, 95% CI [0.083, 1.271], *p* = .012, *d* = 0.52. The two-way interaction between age group and delay confirmed that this age-related decline in performance from the young to the middle-aged participants was generally greater for the STM as opposed to the LTM task when averaged across retrieval success and precision metrics, *t*(122) = −2.31, estimate = −.407, [−0.757, −0.058], *p* = .023. In other words and as shown by estimated marginal means in pairwise comparisons, middle-aged and younger adults performed similarly in terms of retrieval success on the LTM task, *t*(122) = −1.31, estimate = −.303, [−0.852, 0.245], *p* = .392, *d* = −0.24, but younger adults had higher precision for object locations compared to middle-aged adults in the LTM task, *t*(122) = 3.64, estimate = 0.775, [0.270, 1.280], *p* = .001, *d* = 0.66. For STM, middle-aged adults had both significantly lower precision, *t*(122) = 3.05, estimate = 0.625, [0.138, 1.113], *p* = .08, *d* = 0.55, and made more guessing responses, *t*(122) = 3.02, estimate = 0.661, [0.142, 1.181], *p* = .009, *d* = 0.55. For the comparison of old and middle-aged adults, the magnitude of the age effect was not statistically significantly affected by delay or memory processes; all interactions, *t*(122) < 2, *p* > .1, *d* < |.3|. Pairwise tests showed these groups were similar in performance; all *t* < 2.5, *p* > .1, *d* < .3, except for STM precision, which further declined in the older group: *t*(122) = 3.51, estimate = 0.632, [0.205, 1.060], *p* = .002, *d* = 0.64.[Fig fig4]

When using age as a continuous variable in a sensitivity analysis, a mixed linear model with the participant as a random factor, an interaction between age and memory process indicated a steeper decline in precision as opposed to retrieval success, β = −.348, 95% CI [−0.559, −0.138], *t*(369) = −3.22, *p* = .001, *d* = −0.34. There was also an interaction between age and delay, with STM evincing greater age-related decline than LTM performance, β = −.268, [−0.478, −0.057], *t*(369)=−2.48, *p* = .014, *d* = −0.26. Estimated marginal means for this model are shown in Figure 4B.

We further provide evidence of age effects on precision in sensitivity analyses controlling for retrieval success (see Section 2.3 in the online supplemental materials) and using an adjusted version of the precision metric based on a cutoff for guess trials derived from modeling across all participants (Section 2.4; Gellersen, Coughlan, et al., 2021; Richter et al., 2016), demonstrating the robustness of our findings.

### Are Memory Precision Metrics More Sensitive to Age Effects Than Standard Neuropsychological Tasks?

**Figure 5 fig5:**
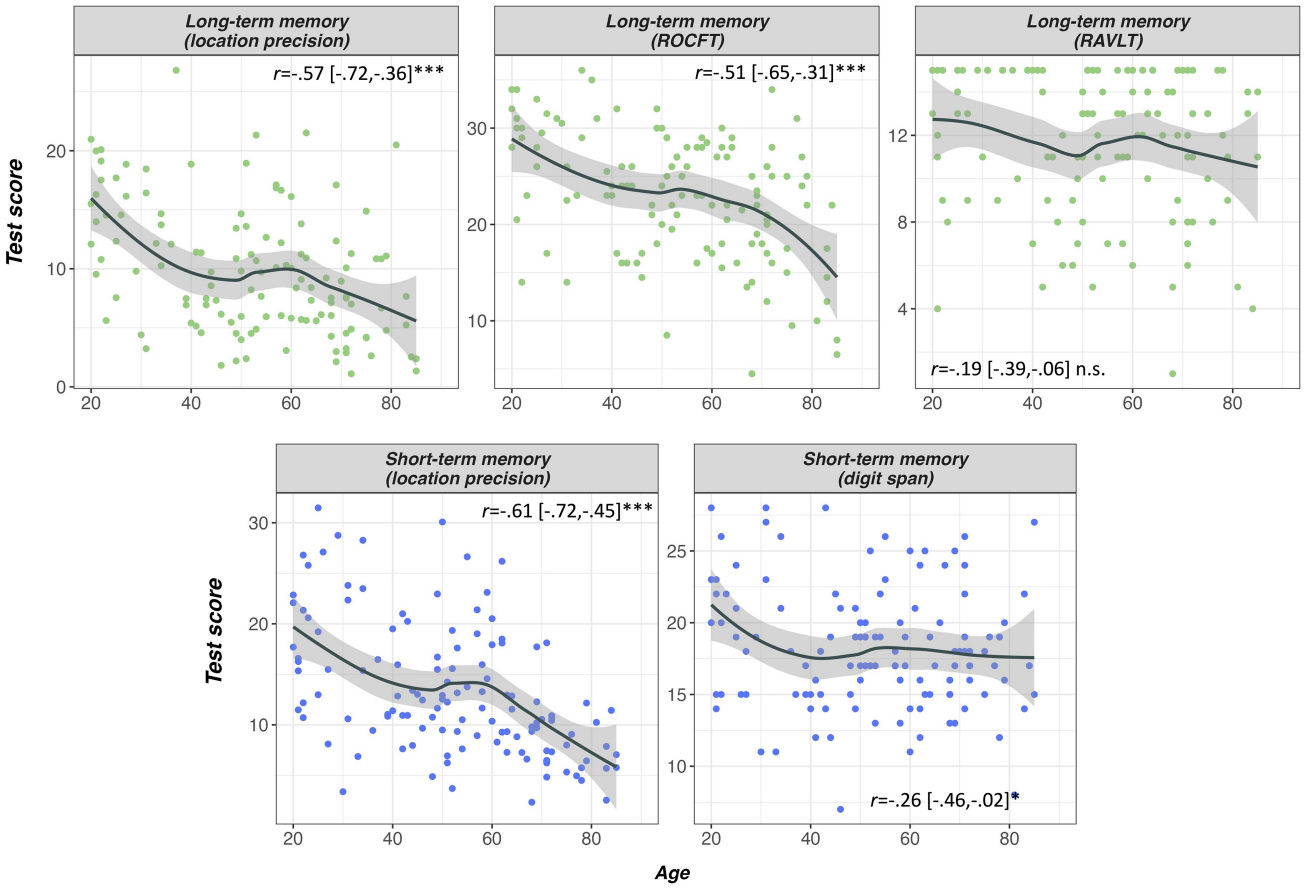
Comparison of Age Effects on Memory Precision Measures and Neuropsychological Test Performance *Note*. Trend lines are modeled with a loess function. Pearson’s *r* values were obtained from a bootstrapping procedure with 10,000 samples after removing extreme outliers with absolute scores larger than *z* = 3. Precision is expressed based on the concentration parameter κ. Abbreviations: *n.s.* = not significant; RAVLT = Rey auditory verbal learning test; ROCFT = Rey–Osterrieth complex figure test. See the online article for the color version of this figure.

We hypothesized that memory precision could uncover subtle age deficits not observable when using standard neuropsychological tasks. We, therefore, compared age effects on memory precision and performance on neuropsychological tests, focusing on a contrast of LTM κ with delayed memory scores and of STM κ with digit span (see Figure 5). We conducted mixed ANOVAs with age group as between-subjects factor, task as within-subjects factor, and years of education and sex as covariates. As expected, the comparison of LTM precision and delayed RAVLT scores showed not only a main effect of age group, *F*(1, 102) = 13.41, *p* < .001, η_*p*_^2^ = .21, but also an interaction of group and task, *F*(1, 102) = 6.18, *p* = .003, η_*p*_^2^ = .11. Post hoc tests showed that age-related declines in LTM precision were significantly larger than those on verbal learning in the middle-aged group, *t*(102) = 2.47, estimate = 0.741 95% CI [0.145, 1.336], *p* = .015, *d* = 0.49), whereas the decline from mid- to late life was equivalent across tasks, *t*(102) = 1.16, estimate = 0.289, [−0.205, 0.783], *p* = .248, *d* = 0.23. This interaction suggests that memory precision can detect small age-related changes in memory performance that are not apparent when using a standard neuropsychological test without demands on memory fidelity. In contrast, when a detailed visual representation is required, such as in the ROCFT, age deficits were equivalent to those in memory precision as shown by the absence of a task by age group interaction, *F*(1, 103) = .64, *p* = .530, η_*p*_^2^ = .01.[Fig fig5]

There was also an interaction of task and age group in the comparison of STM precision and digit span, *F*(1, 120) = 8.42, *p* < .001, η_*p*_^2^ = .12. In contrast to the LTM task, for STM, middle-aged adults were similarly impaired in both digit span and precision, *t*(120) = 1.35, estimate = 0.325, 95% CI [−0.153, 0.804], *p* = .180, *d* = 0.25. Subsequent age-related STM decline between mid- and late life was significantly greater for STM precision as opposed to digit span tasks, *t*(120) = 2.96, estimate = 0.609, [0.202, 1.017], *p* = .004, *d* = 0.54.

We further provide a full overview of age effects across all cognitive measures of interest derived from a bootstrapping procedure in Section 4 (Figure S5) in the online supplemental materials. The largest age effect sizes are found for cognitive measures that assess the fidelity of perceptual and mnemonic representations, both for single objects and for the spatial context of objects.

### Is Memory Precision Derived From Mixture Modeling More Sensitive Than Mean Absolute Error?

The model comparing mixture modeling with a model-free metric of object-location fidelity found a significant main effect of age group, *F*(2, 122) = 21.23, *p* < .001, η_*p*_^2^ = .26, a significant two-way interaction between age group and method, *F*(2, 122) = 3.56, *p* = .031, η_*p*_^2^ = .06, and a three-way interaction between age group, delay and method (mixture modeling with κ vs. mean absolute error), *F*(2, 122) = 4.06, *p* = .020, η_*p*_^2^ = .06. All other effects were nonsignificant (*F* < 1.96, *p* > .14, η_*p*_^2^<.04). Following up on these interactions with contrasts of age group differences as a function of method and delay, we found specifically for the LTM task that the precision memory metric derived from mixture modeling was significantly more sensitive to memory fidelity differences, *t*(122) = 2.84, estimate = 0.882, 95% CI [0.046, 1.01], *p* = .014, *d* = 0.51: middle-aged adults had significantly lower LTM precision than younger adults, *t*(122) = 3.64, estimate = 0.775, [0.270, 1.280], *p* = .001, *d* = 0.66, whereas the mean absolute error did not find a difference between these two age groups, *t*(122) = −.25, estimate = −.057, [−0.601, 0.487], *p* = .962, *d* = −0.05. For all other age group comparisons, the choice of method did not affect the magnitude of group differences described above (all *t* < 1.5, *p* > .25, *d* = 0.15), suggesting that the use of the precision metric in the STM task did not reveal greater performance differences between younger and middle-aged adults than using mean absolute error. These effects are shown in Figure 4C.

A sensitivity analysis on mean absolute errors showed a main effect of age group, *F*(2, 122) = 7.29, *p* = .001, η_*p*_^2^ = .11, and delay, *F*(1, 122) = 36.34, *p* < .001, η_*p*_^2^ = .23, with mean localization errors being smaller in the STM task, but no interaction between age group and delay, *F*(2, 122) = 2.28, *p* = .107, η_*p*_^2^ = .04. Younger and middle-aged adults did not differ in mean localization error on the LTM task, *t*(122) = −.25, estimate = −.848, 95% CI [−8.98, 7.28], *p* = .967, *d* = −0.05, whereas middle-aged adults did commit larger localization errors in the STM task, *t*(122) = 3.23, estimate = 5.878, [1.562, 10.194], *p* = .004, *d* = 0.59. In contrast, localization errors in older compared to middle-aged adults were only larger in the long-, *t*(122) = 2.43, estimate = 7.300, [0.161, 10.194], *p* = .044, *d* = 0.44, but not the STM task, *t*(122) = 2.16, estimate = 3.449, [−0.341, 7.238], *p* = .082, *d* = 0.39. This finding, together with the above analyses, demonstrates that a precision metric can reveal age effects in midlife even when a categorical performance measure cannot. Importantly, the precision metric is shown to be more sensitive to subtle changes in LTM fidelity than mean absolute error measures.

Finally, we examined the relationship between mean error, precision, and retrieval success by comparing correlation coefficients using Steiger’s *z*-test with the R corcor package (Diedenhofen & Musch, 2015; Steiger, 1980). The correlation between mean absolute error and retrieval success (*pT*) was significantly higher than the correlation between mean absolute error and precision (κ), both for the STM (*z* = −7.15, *p* < .001) and the LTM task (*z* = −7.83, *p* < .001; in line with Harlow & Donaldson, 2013). These findings suggest that mean absolute error measures are strongly associated with the accessibility of the memory trace and may therefore obscure the fidelity of actually retrieved memory content (see also Section 5 and Figure S6 in the online supplemental materials).

### Is Aging Associated With a Reduction in the Holistic Retrieval of High-Fidelity Intraitem and Spatial Information?

We hypothesized that correctly remembering an item’s identity in the mnemonic discrimination task would be associated with higher fidelity of that same item’s spatial information as measured using mean absolute error. We further expected this association to be weakened with age, reflecting a reduction in holistic memory retrieval. A mixed linear model found that incorrectly identifying an item on a given mnemonic discrimination trial was associated with poorer object-location memory fidelity as measured using mean absolute localization error, *F*(1, 18,830) = 147.04, *p* < .001. This effect of item identification was present regardless of delay, β = −.237, 95% CI [−.302, −.173], *t*(18,836)=−7.20, *p* < .001, *d* = −0.11, but was larger for the long-term compared to short-term task, β = .115, [0.030, 0.202], *t*(18,805) = 2.63, *p* = .009, *d* = 0.04. This effect is shown in Figure 4D. The interaction between age group and item identification was not significant, *F*(1,18,821) = .44, *p* = .647, suggesting that the effect of item identification on trial-by-trial variability in localization error was equivalent across age groups.

During the revision process, reviewers raised multiple questions about the trial-by-trial nature of memory performance. We therefore conducted the following exploratory analyses using mixed linear models. First, we determined whether the distance between a target and its nontarget neighbors is associated with localization and mnemonic discrimination performance. This may occur due to increased interference between objects in greater proximity to one another. There was no such relationship for object-location errors (*F* *<* .5, *p* > .5). However, for item recognition, there was an interaction between task and distance, which revealed that only in the STM task, mnemonic discrimination accuracy was higher in trials with a greater distance between target and nontarget items, *F*(1, 3,833) = 26.17, *p* < .001.

We also tested for memory dependencies of spatial information within a display. If object-location errors are independent of display, the similarity between placement of a target and a nontarget item should not be affected by whether the other item was part of the same encoding display. In a mixed model with target location error as outcome, we therefore used either the mean location error of the other two nontarget items of the same display or an error randomly sampled from among all trials for a given participant as predictor. This allowed us to isolate within-display effects while controlling for overall memory performance. A three-way interaction between age group, other-item localization error, and type of other item (within-display vs. any trial) found that localization errors of other items on the same display were a better predictor of target localization accuracy than localization errors of items on other displays. Importantly, this effect is diminished with age, *F*(1, 18,637) = 7.63, *p* < .001. There was no effect of test order (*F* < 1, *p* > .8).

One reviewer suggested that an alternative explanation for our finding of the trial-by-trial association between item-level and spatial information may be the effects of encoding for a display as a whole. We, therefore, assessed within-object binding of item and spatial information while accounting for memory for items on the same display. Given that all three items on a given display were tested on the LTM but not the STM task, this analysis was only conducted for the long-term precision paradigm. We found an interaction between item recognition accuracy and item status (target, nontarget), indicating a greater dependency of item-level and spatial information for the same as opposed to other objects on a given display, *F*(1, 18,626) = 16.64, *p* < .001. This lends further support to holistic retrieval suggesting that the effect cannot solely be explained based on overall display-level encoding success.

### Which Cognitive Factors Underpin Individual Differences in Object-Location Memory Fidelity Across the Lifespan?

**Figure 6 fig6:**
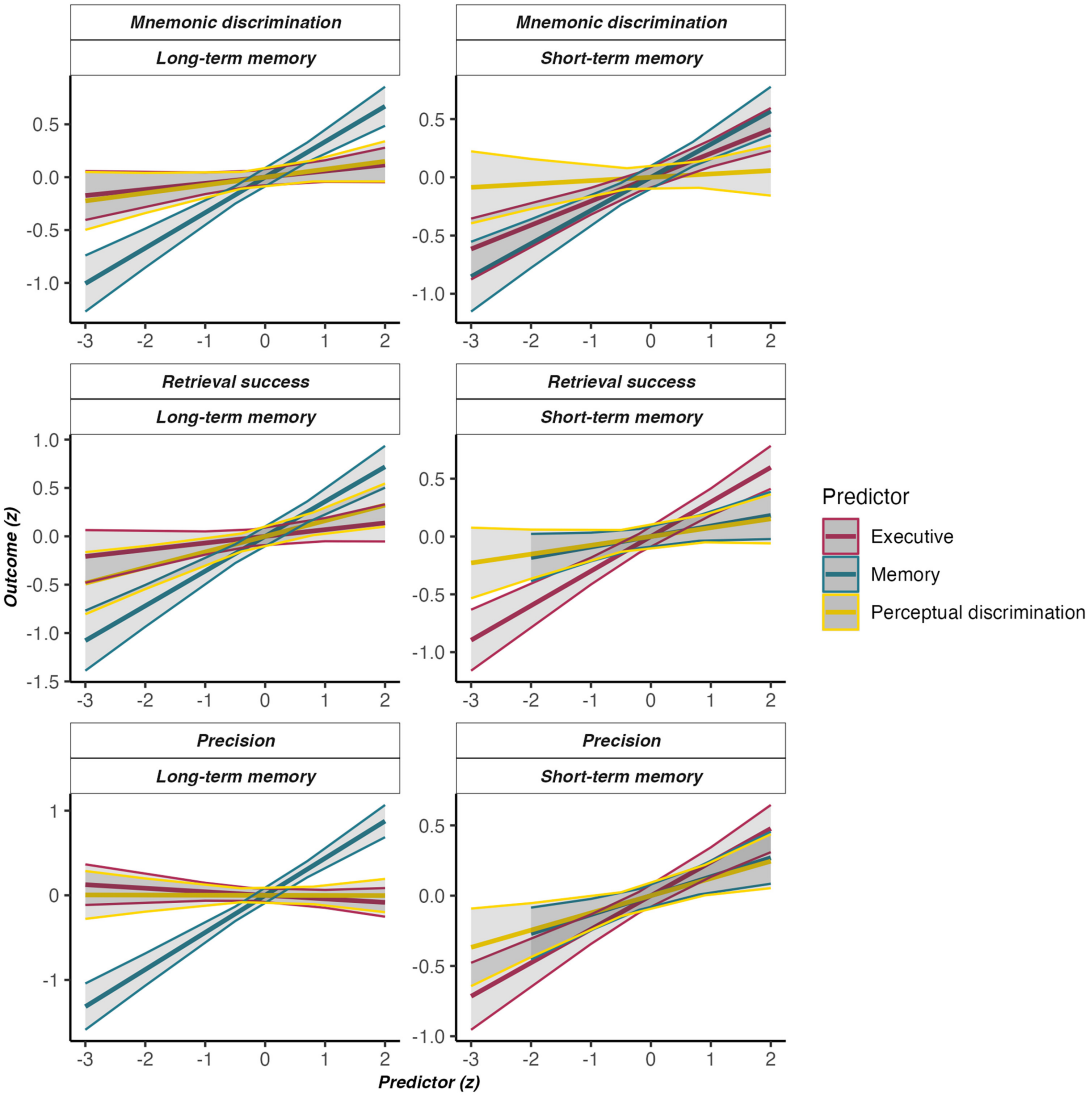
Overview of Independent Contributions of Perceptual, Memory, and Executive Scores to Individual Differences in Memory Performance Across Tasks of Interest *Note*. Trend lines represent the fit and standard error for the respective predictor as identified in multiple linear regression analyses containing age, education, executive functions, neuropsychological memory scores, and high-ambiguity object perceptual discrimination as independent variables. All variables are normalized using *z*-scoring. See the online article for the color version of this figure.

We aimed to identify predictors of retrieval success and precision in the localization task and mnemonic discrimination for object recognition. Predictors of interest were the perceptual discrimination task known to be a proxy of perirhinal cortex integrity (Barense et al., 2007), an executive functioning composite derived from standard neuropsychological tests associated with PFC integrity (Aleman & van’t Wout, 2008; Phelps et al., 1997; Zakzanis et al., 2005), and a delayed memory composite (from the ROCFT and RAVLT). Although these tests are not process pure, both tasks are commonly used indicators for LTM retention and are associated with hippocampal integrity (Bohbot et al., 1998; Trelle et al., 2017). Although prefrontal cortical regions of course also play an important role in memory retrieval, in cases where poorer scores on the delayed memory composite are to a greater extent due to PFC than hippocampal dysfunction, we would expect the executive functioning score to be a superior predictor for performance in the dependent variable of interest in our multiple regression analysis. Due to shared variance, the memory composite score may no longer be retained in such a model. It is therefore the relative differences in the contribution of our predictors to individual differences in memory fidelity that are of particular interest. An overview of step-by-step model comparisons based on the Akaike Information Criterion (AIC) can be found in Section 6 in the online supplemental materials. Figure 6 shows the independent effects of perceptual discrimination, neuropsychological memory performance, and executive functions for each model.[Fig fig6]

Individual differences in short-term mnemonic discrimination could best be explained by performance on executive, β = .205, 95% CI [0.041, 0.368], *t*(117) = 2.48, *p* = .015, *f*^2^ = .08, and memory composite scores, β = .293, [0.113, 0.473], *t*(117) = 3.22, *p* = .002, *f*^2^ = .09; model adjusted *R*^2^ = .22. In contrast, the best fitting model for long-term mnemonic discrimination only included the neuropsychological memory composite score, β = .370, [0.213, 0.526], *t*(118) = 4.67, *p* < .001, *f*^2^ = .18; model adjusted *R*^2^ = .39. Perceptual discrimination scores and STM mnemonic discrimination on their own were also significant predictors, while executive functions were not (see Section 6 in the online supplemental materials).

For STM retrieval success, the best model included executive functioning as the sole predictor of interindividual differences, β = .320, 95% CI [0.158, 0.483], *t*(117) = 3.90, *p* < .001, *f*^2^ = .13; model adjusted *R*^2^ = .21. For the corresponding LTM model, perceptual discrimination on its own was a significant predictor of performance, β = .280, [0.074, 0.486], *t*(116) = 2.69, *p* = .008, *f*^2^ = .06; model adjusted *R*^2^ = .07. Although this effect was no longer significant once the memory composite was included, β = .150, [−0.049, 0.350], *t*(115) = 1.49, *p* = .139, *f*^2^ = .07, according to the AIC both predictors were chosen for the final model; memory composite: β = .398, [0.210, 0.586], *t*(115) = 4.19, *p* < .001, *f*^2^ = .15; model adjusted *R*^2^ = .17.

For memory precision, both the executive functioning, β = .236, 95% CI [0.088, 0.383], *t*(117) = 3.16, *p* = .002, *f*^2^ = .11, and memory composite scores were selected for the STM model, β = .179, [0.012, 0.341], *t*(116) = 2.19, *p* = .030, *f*^2^ = .04; model adjusted *R*^2^ = .37, whereas the model for LTM only included the memory composite, β = .429, [0.268, 0.591], *t*(116) = 5.26, *p* < .001, *f*^2^ = .24; model adjusted *R*^2^ = .36. The STM precision predictor for LTM precision was only at trend level (see Section 6 in the online supplemental materials).

In addition to the neuropsychological memory composite, exploratory analyses shown in Section 6 in the online supplemental materials also found object mnemonic discrimination scores in the LTM task to be significant predictors of retrieval success and precision, while the same was not true for the STM task. All effects reported above held even after controlling the models for memory precision for the respective measures for retrieval success and when controlling the LTM precision model for STM κ.

## Discussion

Here we demonstrate that a loss of representational fidelity is a ubiquitous characteristic of normal cognitive aging throughout the lifespan in the domains of complex perception, working memory, and LTM. Importantly, we show that fidelity metrics are capable of identifying subtle declines in LTM function that emerge in midlife, even when other commonly used memory and cognitive tests show no such age effects. Specifically, LTM and STM precision measures could uncover age group differences undetectable to neuropsychological tests of delayed verbal learning and digit span, respectively. Our findings further suggest that a mixture modeling approach to estimate memory precision for successfully retrieved information is more sensitive to subtle differences in memory fidelity than mean localization errors across all trials. Moreover, negative age effects on the precision of object-location binding were greater for STM as opposed to LTM. LTM precision significantly declined between younger and middle-aged adults but the further reduction in older adults was smaller and not statistically significant, whereas STM precision declined consistently across age groups. Younger adults performed better on object mnemonic discrimination than the older groups, but a further age-related decline from midlife to late life was only observed on the LTM, not the STM version of the task. We also shed light on the relationship between working memory and LTM precision, showing small to moderate associations across the lifespan even if the similarity of stimulus material between these tasks is matched. Finally, we show that the cognitive factors underpinning age effects and interindividual differences in memory precision in STM and LTM, respectively, are at least partially dissociable, with behavioral indices of executive functions (PFC-dependent) being exclusively identified as predictors for STM regardless of whether fine or coarse-grained representations were taxed, while the delayed memory composite score (a proxy for hippocampal processes) were included in both models for STM and LTM when high-fidelity mnemonic representations were required.

### Age-Related Declines in Perceptual and Mnemonic Fidelity

Our findings demonstrate that previously identified age-related impairments in complex perceptual processes (Burke et al., 2011, 2012; Devlin & Price, 2007; Ryan et al., 2012) and mnemonic discrimination in old age are present in midlife (Güsten et al., 2021; Nauer et al., 2020; Stark et al., 2013, 2019; but see Samrani et al., 2022) and replicate findings of poorer memory fidelity for object and spatial information in older adults (Reagh et al., 2016; Stark et al., 2019). We further expand upon prior research by demonstrating that mnemonic discrimination is even impaired when taxed at short delays without interfering trials. Our results suggest that the ability to form viewpoint-invariant high-fidelity representations of complex visual stimuli and to counteract feature interference may be key to understanding which cognitive processes will show early detrimental age effects.

We provide the first evidence that LTM precision of relational binding underpinning source memory is reduced in midlife even if the probability of successful retrieval is unimpaired. Having used a mixture modeling approach for the estimation of memory precision afforded us greater sensitivity to subtle memory differences, potentially explaining why a similar continuous recall test using mean absolute errors found no age effects in midlife (Čepukaitytė et al., 2023). Our findings are in line with those in studies contrasting younger and older adults (Korkki et al., 2020; Nilakantan et al., 2018; Rhodes et al., 2020) pointing to a decline in representational quality as one of the earliest signs of age-related episodic memory deficits. This is further supported by the finding that the ROCFT, which requires participants to recreate details of an abstract image, was the only LTM neuropsychological test in which middle-aged adults performed worse than younger adults. The ROCFT is most similar to our paradigm in its mnemonic demands on high-fidelity representations, while other measures included in the ACE or the RAVLT do not tax representational quality to the same extent.

These results may explain mixed findings with respect to LTM declines in midlife (Cansino, 2009; Cansino et al., 2012; LaPlume et al., 2022; Park & Festini, 2016). A reduction in representational fidelity would explain why middle-aged adults, similarly to their older counterparts, are impaired relative to younger adults when tasks involve high levels of perceptual and semantic interference (Güsten et al., 2021; Stark et al., 2013; Williams et al., 2019, 2020), as demonstrated consistently throughout this study on all tasks reliant on detailed perceptual and mnemonic representations. Because prior studies have relied on categorical responding, it was previously not clear whether these types of memory deficits were driven by reduced memory accessibility or fidelity. Our data suggest that middle-aged adults may be similarly capable of accessing the contents of their LTM than younger adults, with the caveat that retrieved representations are more coarse-grained. These findings therefore lend further support to the proposal that the fidelity of memory is relatively more sensitive to aging than the probability of successful recall. We show mixture modeling to be a powerful tool to uncover these subtle age effects due to its capability to separate effects of memory accessibility and fidelity.

We also replicate prior findings showing that aging is associated with reduced precision of working memory, again demonstrating that this effect emerges in midlife (Korkki et al., 2020; Manga et al., 2021; Mitchell & Cusack, 2018; Noack et al., 2012; Peich et al., 2013; Pertzov et al., 2015; Rhodes et al., 2020). However, our finding of poorer working memory retrieval success in middle-aged and older adults deviates from prior studies using mixture modeling on STM data (Korkki et al., 2020; Peich et al., 2013; Rhodes et al., 2020). Given that participants were asked to both recall object positions and maintain a high-fidelity representation of intraobject details, the increase in memory load during the maintenance period may have affected middle-aged and older adults disproportionately compared to younger adults, resulting in more instances of forgetting with age (Kwon et al., 2016; Peich et al., 2013). Performance in the two older groups resembled the effects of an increase in memory load or maintenance period for younger adults shown in previous studies (Bays & Husain, 2008; Bays et al., 2009, 2011; Gorgoraptis et al., 2011). Our data show that at this set size, working memory resources available to middle-aged and older adults are not only insufficient to maintain high-precision representations, but also incapable of counteracting complete forgetting of spatial contextual information. Interestingly, the decline in STM precision from mid- to late life was steeper than that measured with the standard digit span task. Again, this suggests an added benefit of investigating age-related changes in working memory with measures capable of indexing representational fidelity.

Moreover, we demonstrate a strong association between the veridical retrieval of item and spatial information in both STM and LTM. That is, correctly discriminating between a target and its corresponding lure on a given trial was associated with a closer match between the initial location of the object in question and the placement of that object during retrieval. Prior studies typically only investigated either STM or LTM in isolation and did not use comparable stimulus materials between these tasks, making it difficult to examine the effect of delay and degree of feature interference on holistic retrieval (Cooper & Ritchey, 2019; Grande et al., 2019; Horner et al., 2015; Ngo et al., 2021). We provide evidence that this association between different types of item features is stronger in LTM as opposed to STM. We also extend prior findings by demonstrating that correctly remembering item information is not only associated with the recall of the gist but also the fidelity of spatial information. Importantly, associations between object details and object-location fidelity were significantly stronger within a given object than between different objects of the same encoding display. This suggests that the effects of encoding the display as a whole are not a sufficient explanation for the within-display dependency of the fidelity of item and spatial information. These findings may reflect the episodic nature of LTM whereby the retrieval of features presented in the same context may lead to cortical reinstatement of associated features following hippocampal pattern completion, which promotes holistic retrieval (Barry & Maguire, 2019; Grande et al., 2019; Horner et al., 2015; Horner & Burgess, 2014). Intriguingly, we did not find evidence for our hypothesis of the diminished within-object item and location binding across the lifespan, but our exploratory analysis for the LTM task revealed that within-event dependency of spatial information of the three objects was weaker with higher age. This suggests that at least some age-related reduction in holistic retrieval may have occurred for between- but not within-item information. These findings are partially in line with prior studies, which may be due to differences in the experimental paradigm given that other memory paradigms asked older adults to bind together more individual features (for scenes, objects, and persons or temporal order), which may have increased representational complexity and memory load (Cheke, 2016; Ngo & Newcombe, 2021).

### Methodological Considerations and the Sensitivity of Mixture Modeling

When conducting our analysis on mean absolute error instead of the model-derived precision metric, we did not find differences in LTM performance between age groups. These findings mirror those of Nilakantan et al. (2018) who also found that the age effect in their sample of older adults was specific to a model-informed precision metric. This is likely due to the fact that the mean absolute error incorporates data from guess trials, while the mixture modeling procedure exclusively assesses memory fidelity for trials in which successful recall did take place. In line with this notion, correlations between retrieval success and mean errors in the object-location tasks were significantly greater than correlations between mean errors and precision, suggesting that mean target-response errors are relatively more reflective of the ability to retrieve any trial-relevant information from memory. While mean absolute error metrics do provide important insight into memory fidelity, it is important to note that the interpretation of findings from these studies may be relatively more influenced by the frequency of guessing (Čepukaitytė et al., 2023; Zokaei, Čepukaitytė, et al., 2019; Zokaei, Nour, et al., 2019) and reliance on this model-free metric may have prompted us and Nilakantan et al. (2018) to conclude that object-location binding may be relatively unaffected by healthy aging. The mixture model is thought to reflect the thresholded nature of hippocampal pattern completion that is key during the reinstatement of object-location information from LTM (Horner & Burgess, 2014; Ngo et al., 2021; Vieweg et al., 2019). The precision metric as opposed to the mean absolute error may better capture this all-or-some nature of hippocampal processes in LTM (Harlow & Donaldson, 2013), with reinstatement being successful in some trials, with varying degrees of fidelity, while entirely failing in others (Elfman et al., 2014; Harlow & Donaldson, 2013; Norman, 2010; Yonelinas, 1994; Yonelinas et al., 1998).

Surprisingly, we did not identify a significant difference in swap errors between age groups or a meaningful contribution to performance in working memory more generally across participants, even though previous studies consistently demonstrate that misbinding is common in these types of tasks (Bays et al., 2011; Peich et al., 2013; Pertzov et al., 2015; Zokaei et al., 2014; Zokaei, Nour, et al., 2019). There are important methodological differences between previous studies and the present investigation, which may explain this discrepancy. The stimuli used in our task were highly distinct everyday objects as opposed to abstract fractals (Pertzov et al., 2015; Zokaei, Čepukaitytė, et al., 2019; Zokaei, Nour, et al., 2019) or bars previously used (Bays et al., 2011; Peich et al., 2013). As a result, participants may have been able to draw on verbal rehearsal strategies or activation of semantic representations from LTM (Kowialiewski et al., 2021; Rose et al., 2010) and benefited from richer perceptual representations (Veldsman et al., 2017), therefore reducing the likelihood of swap errors. Although this design introduces the potential of age differences in semantic encoding and grouping strategies (Craik & Rose, 2012; O’Donnell et al., 2018), a design involving semantically meaningful stimuli has several advantages: (a) it is more likely to reduce age deficits (Kirchhoff et al., 2012), therefore making our findings of age-related deficits in short-term memory precision in midlife even more striking, (b) has greater ecological validity than prior studies (Mitchell & Cusack, 2018; Peich et al., 2013; Pertzov et al., 2015), and (c) allows for the use of similar stimuli and encoding demands in both STM and LTM tasks (Korkki et al., 2020; Rhodes et al., 2020).

### Mechanisms of Age-Related Declines in Mnemonic and Representational Fidelity

Here we chose three measures of cognitive functions we hypothesized to explain interindividual differences in memory fidelity. First, as aging results in less differentiated neuronal representations throughout the visual hierarchy (Burke et al., 2018; Carp et al., 2011; Johnson et al., 2021; Koen & Rugg, 2019; Li et al., 2001, 2005; Park et al., 2010; Ryan et al., 2012), we used the high-ambiguity perceptual discrimination task to index the integrity of perirhinal processes needed for the formation of complex representations robust to feature interference (Barense et al., 2007). Second, standard neuropsychological tasks were used to derive a composite executive functioning score using tasks that are known to heavily rely on the integrity of prefrontal cortical regions (Foster et al., 2020; Gellersen, Trelle, et al., 2021). Third, we calculated a delayed memory composite score from tasks that are typically used in neuropsychological assessments to index hippocampal functions (Trelle et al., 2017). Although these tasks are not process pure, their use as predictors in the same model can still provide insights into their differential contribution of more PFC- and hippocampal-dependent processes on individual differences in the outcomes of interest. We consistently show that individual variability in STM performance metrics was associated with executive functioning. In contrast, tasks of hippocampal integrity were associated with all LTM scores and the two STM fidelity measures, but not the STM gist-based metric. Moreover, the association between our STM- and LTM measures was strong for mnemonic discrimination only, but moderate for retrieval success and precision measures despite highly similar stimulus material and encoding conditions. These findings suggest that although working and LTM may share an upper bound of representational precision due to the inherent properties of the visual system (Brady et al., 2013), the factors underpinning individual variability in the successful maintenance and retrieval of high-fidelity memories over short and long delays may be partially dissociable.

In line with our hypothesis, perceptual object discrimination could explain interindividual variability in mnemonic discrimination suggesting that the fidelity of object representations in the perceptual domain may be inherited by corresponding memory representations. Surprisingly though, this association was only present for long delays, as previously shown in a sample of older adults (Gellersen, Trelle, et al., 2021). A potential explanation for this result may be that declines in representational quality are most detrimental at longer study-test delays given higher feature interference, while shorter delays may place fewer demands on the formation of holistic, unique stimulus representations. Our findings of relatively greater age effects on long- as opposed to short-term mnemonic discrimination are in line with this proposal.

Finally, executive functions were only predictive of individual variability on STM scores, suggesting that working memory, processing speed, and inhibitory control were key in guarding memory representations from interference over short delays but could not account for age-related declines in memory fidelity over longer delays. When less encoding time is provided and no LTM representations need to be formed, task performance may be more reflective of the involvement of frontoparietal and attention control networks to maintain information in active storage (Baddeley, 2003; Suzuki et al., 2018; van Asselen et al., 2009). In contrast, a loss of representational fidelity in the perceptual domain and hippocampal failures in pattern completion may be the more decisive factor contributing to age-related impairments in mnemonic discrimination and object-location precision in LTM where feature interference is significantly greater (Clark et al., 2017; Korkki et al., 2020, 2021; Old & Naveh-Benjamin, 2008; Paleja & Spaniol, 2013; Stark et al., 2010; Wang et al., 2016). Intriguingly, the hippocampal memory composite score was also selected in models for short-term mnemonic discrimination and precision, but not retrieval success. This is in line with the proposal that the hippocampus is required for high-resolution binding of visual information across all cognitive domains (Ekstrom & Yonelinas, 2020; Yonelinas, 2013). Indeed, recent evidence lends support for this view, showing hippocampal lesions to result in declines in the precision but not the frequency of forgetting of visual working memory, suggesting that the hippocampus is not required for the maintenance of coarse-grained representations in STM (Borders et al., 2022). Lastly, although a combination of our neuropsychological predictors could explain interindividual differences in memory fidelity, age effects remained. It is possible that domain-general age effects such as increased neural noise and declines in psychomotor speed may be one mechanism affecting performance in all our tasks (Baltes & Lindenberger, 1997; Novotný et al., 2022).

Lastly, although our results suggest a loss of fidelity for visual representations throughout the lifespan, other forms of representations may be less impacted by age. For instance, a recent study has shown an age-related shift away from visual to semantic information being represented in temporal lobe regions during memory recall (Naspi et al., 2023). Interestingly, only in older adults were these semantic representations associated with higher subjective memory vividness. This is in line with previous findings, which suggest that semantic as opposed to episodic memory is better preserved with age and that older adults can use prior knowledge such as schemas to maintain good memory performance if task demands allow (Castel, 2005; Loaiza et al., 2015). However, our paradigms used stimuli with no to little semantic meaning for the perceptual task, while mnemonic lures only involved changes in the configuration of features or minor details (patterns, colors). These stimuli do not lend themselves readily to a compensation strategy based on semantic representations. As a result, we interpret our findings of reduced memory fidelity as resulting predominantly from reduced quality of visual representations.

### Limitations and Caveats

We did not use a perceptual-motor control task in our study. Given that we included both STM and LTM tasks and a neuropsychological battery, we were forced to prioritize for time. Korkki et al. (2020) previously demonstrated that a perceptual control task did not account for age-related deficits in their analog memory task. We therefore decided to focus on the perceptual discrimination tests to assess age effects on complex perception. In future studies, it may be informative to include tests of visual acuity, which has previously been shown to be correlated with mnemonic discrimination tasks (Davidson et al., 2019; but see Jensen et al., 2023 in a larger sample including the same participants) and may also impact the precision of memory.

We also did not include a recognition memory question for novel foil items, as was done in previous studies (Peich et al., 2013; Pertzov et al., 2015; Rhodes et al., 2020). These studies used foil trials to account for potential forgetting of studied items. Performance for the foil recognition tasks was typically at ceiling, often above 90% even in the older adult group, and Rhodes et al. (2020) report that age differences in location precision were similar regardless of whether a task took into account trials with false identification of foils or not. Even in LTM, novel foil recognition with aging is often unimpaired (Devitt & Schacter, 2016; Stark et al., 2019). Our sample consisted entirely of high-functioning, cognitively healthy individuals. Including a proportion of foil trials in our paradigm would have reduced the number of trials available for estimation of mnemonic discrimination performance and was unlikely to be particularly informative, especially given that we included standard neuropsychological tests that can control for any obvious memory impairments.

Finally, we note the following constraints on generalizability of our findings. Although we took care to use a wide range of participant recruitment channels both online and in person (community groups, notice boards, handing out flyers) and although we did not primarily sample our younger adult group from the University of Cambridge student population, our sample included mostly white participants and participants with higher educational attainment than the average U.K. population (16.5 vs. 13 years; World Economics, 2023). Moreover, it is likely that older adults included in this study are more socially engaged and cognitively healthy than the average senior citizen given that our recruitment channels included community groups and given participants’ ability to travel to the lab unassisted. However, given these constraints, it is even more notable that the memory fidelity metrics could identify subtle age effects in this above average cognitively healthy older adult sample.

### Conclusions

We provide a comprehensive assessment of memory and representational fidelity across the whole lifespan by employing an individual differences approach and including tasks across multiple cognitive domains. We show that performance declines in midlife are consistently observed when detailed and precise representations are required. In contrast, negative age effects on cognitive performance are largely absent when more coarse-grained, gist-based representations are sufficient for perceptual discrimination and LTM recall. Importantly, memory precision identified subtle age-related declines in LTM in middle-aged adults that would be missed by standard neuropsychological memory tasks that define performance based on quantity (e.g., number of recalled words). Declines in representational fidelity may therefore be among the earliest detrimental signs of aging across domains of perception and memory. An approach capable of isolating memory precision from the success of memory recall can uncover subtle age effects on memory that may not otherwise be measurable. In contrast, as STM may be negatively affected at an earlier age, both quantity and quality of to-be-recalled features are reduced from midlife, with precision moving on to decline more steeply into old age.

Moreover, the fidelity of item and spatial information is more tightly bound in long- as opposed to STM suggesting more holistic retrieval in LTM. Importantly, this association between item and spatial information within the same event in LTM could not be fully explained by encoding effects but points to a dependency structure during retrieval. Our findings also suggest that greater proximity to other within-event items may negatively affect mnemonic discrimination accuracy in STM, even if items presented in a given context are not semantically or perceptually similar. This effect was independent of age and may reflect general properties of the human memory system in that increased within-event perceptual load leads to interference that hampers the distinction of highly similar objects.

Finally, the same tasks in STM and LTM were only moderately correlated suggesting partially dissociable mechanisms of interindividual variability in memory fidelity. While executive functions were a strong and consistent predictor of STM performance throughout the lifespan, they did not account for declines in LTM. In contrast, neuropsychological tests of delayed memory, often used as proxies of hippocampal integrity, were associated with memory fidelity of both STM and LTM. Future research should aim to use designs capable of closely matching stimuli and encoding phases for short- and long-term retention tasks while investigating shared and dissociable neural underpinnings of deficits across memory domains. Finally, the present findings provide an interesting avenue for future studies into the use of precision measures in the early detection of memory decline.

## Supplementary Material

10.1037/xge0001476.supp

## Figures and Tables

**Table 1 tbl1:** Sample Demographics

Measure	Younger adults(*N* = 30)	Middle-aged adults(*N* = 50)	Older adults(*N* = 52)
Age	25.23 (5.02)	48.84 (6.32)	70.83 (7.19)
Sex	14F/16M	31F/19M	36F/16M
Education	17.13 (3.04)	16.84 (2.71)	15.87 (3.28)
ACE total (/100)	95.75 (4.20)	95.80 (3.73)	95.69 (3.16)
ACE attention (/18)	17.67 (0.80)	17.53 (0.91)	17.58 (1.00)
ACE memory (/26)	24.77 (1.45)	24.48 (1.99)	24.50 (1.64)
ACE verbal fluency (/14)	12.63 (1.65)	12.79 (1.12)	12.77 (1.35)
ACE language (/26)	24.90 (1.84)	25.20 (1.05)	25.27 (0.82)
ACE visuospatial (/16)	15.68 (0.61)	15.76 (0.61)	15.46 (0.94)
Trails B	68.67 (19.99)	77.06 (26.22)	95.00 (46.92)
Digit span total	20.33 (5.16)	17.63 (3.71)	17.78 (4.24)
ROCFT (copy)	35.78 (0.58)	35.49 (1.42)	35.42 (1.02)
ROCFT (immediate)	27.06 (5.35)	24.05 (5.60)	20.50 (6.45)
ROCFT (delayed)	27.24 (5.92)	23.76 (5.36)	20.27 (6.63)
RAVLT (immediate)	13.19 (2.55)	11.81 (2.86)	11.51 (2.77)
RAVLT (delayed)	12.63 (3.18)	11.43 (3.13)	11.29 (3.42)
*Note*. Mean and (Standard Deviations). Significance levels are shown in the “middle-aged adults” column for the comparison of younger and middle-aged participants and in the “older adults” column for the comparison of middle-aged and older adults. A detailed overview of all age effects is shown in the online supplemental materials. ROCFT = Rey–Osterrieth complex figure test; RAVLT = Rey auditory verbal learning test; ACE = Addenbrooke’s cognitive examination.			

**Table 2 tbl2:** Performance on Precision Memory and Object Discrimination Tasks

Metric	Young	Middle	Old
Object perceptual discrimination *d*′ (low ambiguity)	5.45 (0.37)	5.40 (0.43)	5.21 (0.65)
Object perceptual discrimination *d*′ (high ambiguity)	3.25 (0.656)	2.51 (0.75)	2.07 (0.84)
Object mnemonic discrimination *d*′ (STM)	1.47 (0.37)	1.25 (0.29)	1.12 (0.35)
Object mnemonic discrimination *d*′ (LTM)	1.82 (0.54)	1.54 (0.51)	1.19 (0.39)
Mean absolute error (STM)	20.61 (7.44)	26.61 (8.08)	32.05 (10.82)
Mean absolute error (LTM)	31.45 (17.53)	32.53 (15.81)	38.97 (15.00)
*pT* (STM)	0.89 (0.08)	0.82 (0.10)	0.79 (0.09)
*pT* (LTM)	0.74 (0.22)	0.80 (0.16)	0.70 (0.19)
κ (STM)	18.12 (7.18)	13.74 (5.46)	9.87 (4.83)
κ (LTM)	13.88 (4.88)	9.64 (5.39)	7.86 (4.61)
*Note*. Mnemonic and perceptual discrimination scores are measured in *d*′, mean absolute error in degrees, *pT* refers to the proportion of trials within the von Mises distribution, and κ describes the concentration parameter of the von Mises distribution. The means for model estimates *pT* and κ shown here only include those participants for whom the mixture modeling procedure did not fail to produce reliable model estimates. STM = short-term memory; LTM = long-term memory.
