# Supplementary material for: Demands on Perceptual and Mnemonic Fidelity Are a Key Determinant of Age-Related Cognitive Decline Throughout the Lifespan
Source: J Exp Psychol Gen. 2024 Jan;153(1):200–23. doi: 10.1037/xge0001476 (PMC10795485; doi:10.1037/xge0001476)
Supplement: Supplementary file 1 [file xge0001476.pdf]

Supplementary Material

For "*Demands on perceptual and mnemonic fidelity are a key determinant of age-related cognitive decline throughout the lifespan*" (Gellersen, McMaster, Abdurahman, & Simons, 2023)

Table of Contents

1. Stimulus Selection ***2***

2. Bayesian Mixture Modelling ***4***

2.1 Modelling procedure ***4***

2.2. Group-based model comparisons ***8***

2.3 Controlling for retrieval success ***8***

2.4 Alternative estimation of memory precision ***9***

3. Exploratory Analyses Post-Review ***10***

4. Comparison of all cognitive measures ***12***

5. Associations between precision, retrieval success and mean error ***14***

6. Details of Model Selection for Individual Differences in Memory Fidelity ***15***

7. References ***18***

1. Stimulus Selection

We obtained the majority stimuli from https://konklab.fas.harvard.edu/#. We further collected images through Google search while following the examples provided in the aforementioned image collection. Two objects always belonged to the same class and had the same use as to minimize the influence of semantic differences. Rather, their differences were in configuration of features and changes in colours or patterns. This choice was made to not change the nature of the lure stimuli vis-à-vis that of the target.

To ensure that items were distinguishable, yet similar, each target-lure pair was rated by an independent cohort. This cohort consisted of 25 younger adults with a mean age of 24 years (ranging from 20 to 32), a mean of 15 years in education (ranging from 13 to 18 years), and a female-male ratio of 15/10. Participants were asked how perceptually similar the two exemplars appeared to them on a scale of 1 (lower levels of perceptual similarity) to 5 (higher levels of perceptual similarity). They were told that a rating of 1 corresponds to objects which share a few features but still have several distinct features, whereas a rating of 5 refers to objects being almost identical and being only distinguishable based on minute details. The aim was to prevent the use of stimuli which were so similar as to result in a floor effect for the majority of participants. We therefore excluded all objects with a mean rating above 4. An example of stimulus pairs and their respective ratings and inclusion decisions can be found in Figure S1.

Based on these ratings, object pairs were randomly allocated to the short- or long-term task while ensuring that overall target-lure similarity was matched between the two task formats. There was no significant difference in similarity ratings of objects used in the STM and LTM task, respectively ($p<.05$; mean STM: 2.42±.57; mean LTM: 2.57±.45).

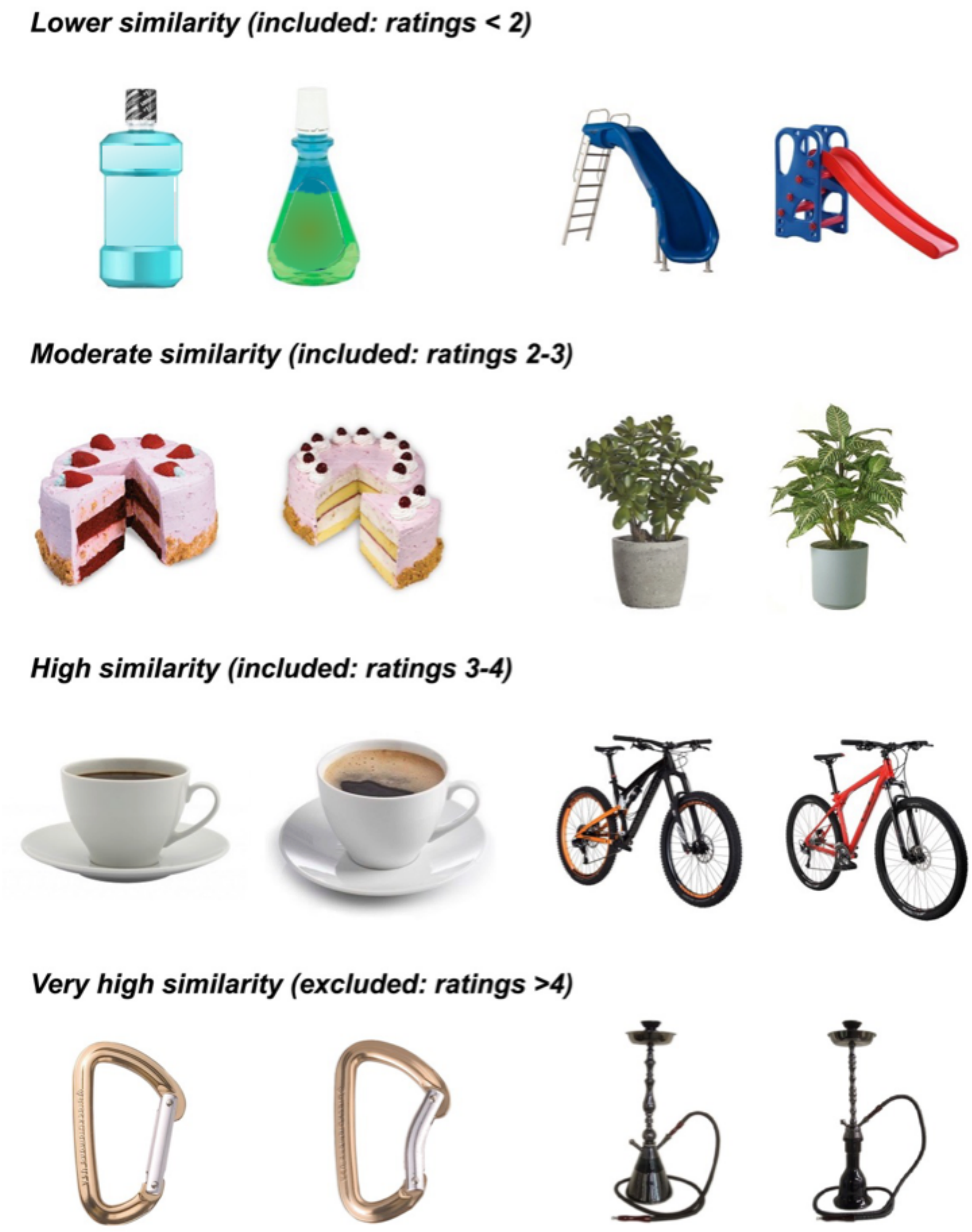

Figure S1. Examples of target-lure stimulus pairs for the mnemonic discrimination task and their subjective similarity ratings derived from an independent cohort of younger adults. Labels on the images of the bottles in the upper panel were obscured due to copyright restrictions.

2. Bayesian Mixture Modelling

2.1 Modelling procedure

The three models fit to the data had the following assumptions (Figure S2A):

1) Model 1 assumes that participants always remember the (approximate) location of all presented items and may simply vary with respect to the trial-by-trial distance between target and responses. In this case, their responses could best be modelled by using a circular Gaussian (von Mises) distribution, the full-width half-maximum of which represents the precision of a subject's responses.
2) Model 2 assumes that participants may recall the location of the target item for some trials with varying degrees of error but in other trials may forget the location of the target object entirely, resorting to guessing (Bays et al., 2011; Zhang & Luck, 2008). In this case, responses can best be characterised in terms of a uniform distribution reflecting random guesses and by a von Mises distribution reflecting the precision of remembered trials. Prior studies in both young and older adults have shown that this model best captures memory responses at longer delays in tasks comparable to the current paradigm (Korkki et al., 2020a; Richter et al., 2016a).
3) Model 3 assumes that in addition to random guesses and correct recalls, participants also mistake the location of a non-target item in the same studied display for that of the target item. In this case they would place the probe object where another object had previously been presented, committing a binding error. Misbinding can be modelled by von Mises distributions centred at the location of one of the two non-target items.

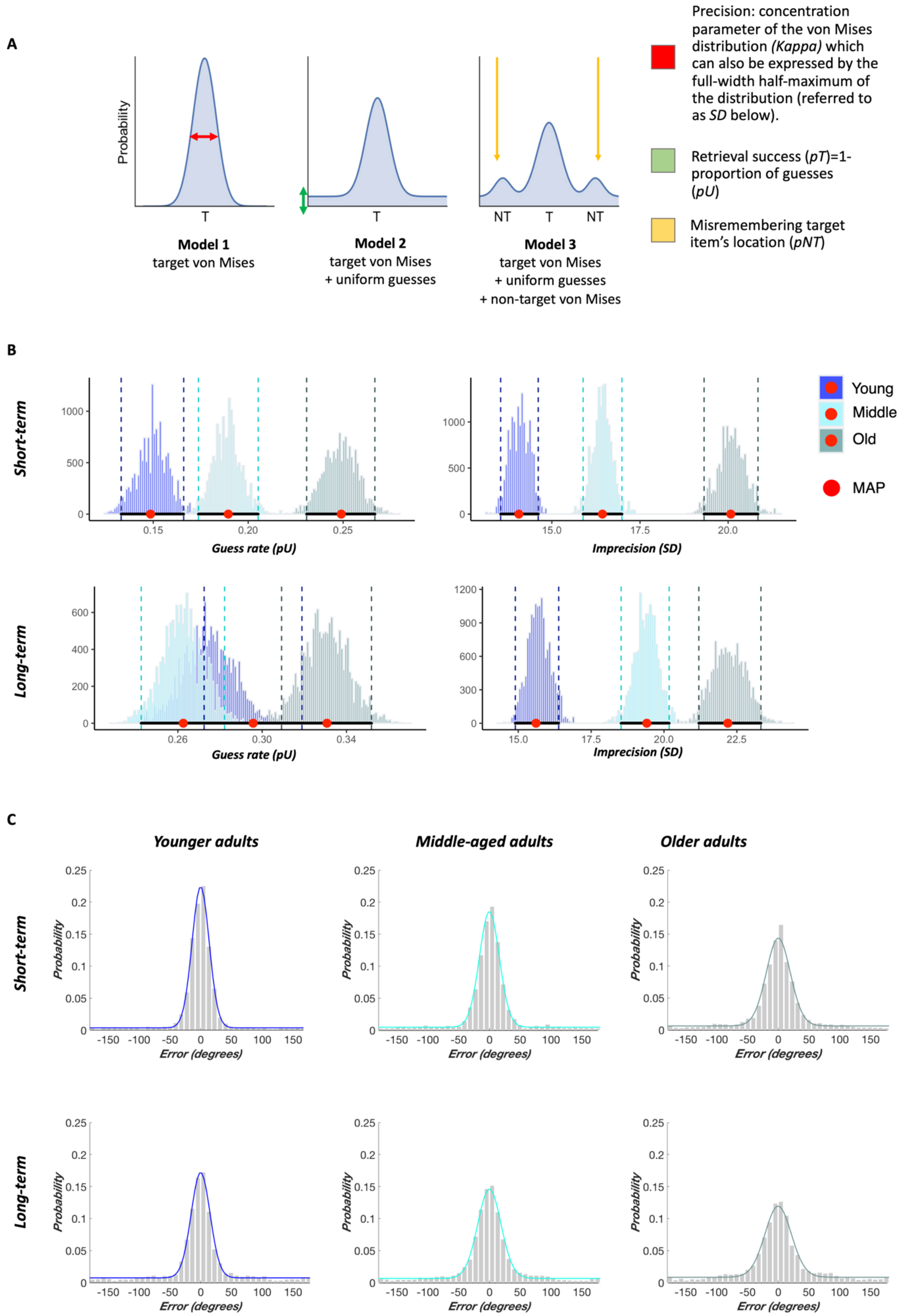

Figure S2. Tested models and results from the mixture modelling approach. A. Proposed models to capture location memory performance. B. Posterior distributions obtained from the Bayesian model fitting procedure for

each age group on short-term and long-term memory data, respectively. Dotted lines represent the upper and lower credible values and the red dot denoted *MAP* represents the maximum *a posteriori* estimate which is taken as the final metric to represent the two model components. C. Standard mixture models best fit localisation error responses, which are here shown across all participants from each of the three age groups, respectively, and by precision memory task (short- vs. long-term). Final chosen model parameters correspond to the respective *MAP* values from B.

A model selection procedure using Deviance Information Criterion favoured Model 2, the standard mixture model, for long-term memory localisation errors and Model 3, the swap model, for short-term memory data (Table S1). However, diagnostic plots showed poor convergence of the swap model (Figure S3) and this model was a poor fit for 56 participants. The average probability of swap errors across participants was 5%, amounting to three misbinding trials across the whole task. We deemed this small number of trials as insufficiently informative to investigate age effects on short-term memory in our task. To date, studies that identified swap models as a better fit for working memory data have used abstract stimuli such as fractals and shapes (Bays et al., 2009; Peich et al., 2013; Pertzov et al., 2015). To our knowledge the only other study on short-term memory modelling which used real-world object stimuli did not find significant instances of misbinding errors even though their study included significantly more trials (120) than the current task (75) and was therefore better suited to detect whether misbinding errors made a meaningful contribution to age effects in their short-term memory task (Korkki et al., 2020b). Given that our study most closely follows their methods, we conducted all further analyses on model estimates derived from the Standard Mixture Model including a uniform and a von Mises distribution. The Bayesian approach determines a probability distribution for each model parameter, showing which parameters could reasonably describe the response data. The posterior distribution for estimates of retrieval success and precision shown in Figure S2B is found using a Markov Chain Monte Carlo algorithm and shows the interval of credible values to describe a given model parameter. The *maximum a posteriori* estimate (the peak of the posterior distribution) is the best estimate for retrieval success and precision, respectively. Figure S2C shows the best fitting model for each age group. We also obtained measures of retrieval success and precision for each subject using the same procedure.

Table S1. Overview of model selection parameters.

Model comparison	STM DIC	STM winning model	LTM DIC	LTM winning model
No guessing model – Standard model	-4380.13	Standard model	-2659.51	Standard model
Swap model – Standard model	-621.21	Swap model	1.12	Standard model

Note. Values show the difference in the Deviance Information Criterion obtained from the Bayesian modelling procedure fitting three candidate models to the target-response error data collapsed across all trials. Negative values favour the left-hand model in the equation.

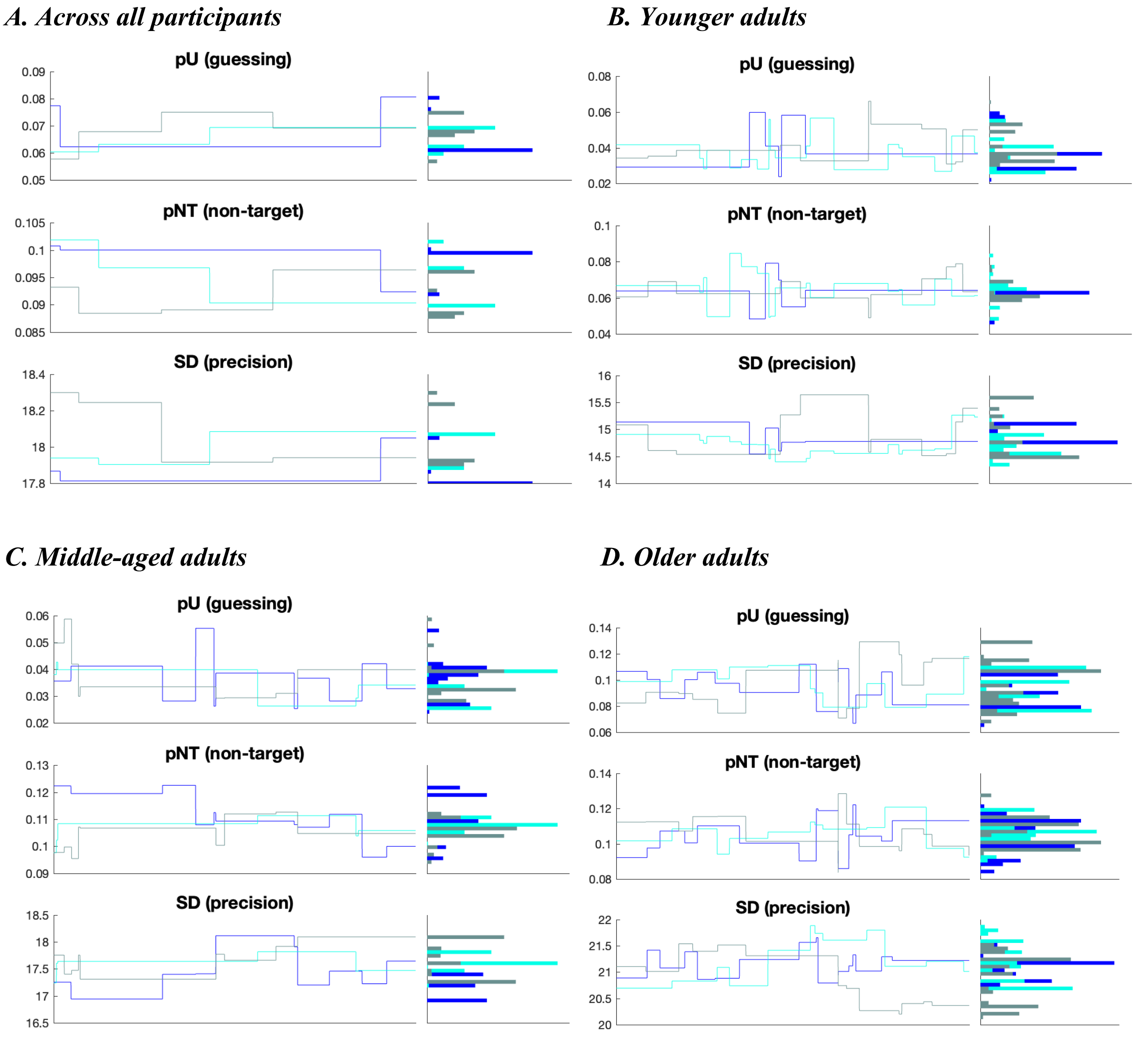

Figure S3. Poor convergence for swap model across all participants and each age group.

2.2. Group-based model comparisons

We compared age groups using mixture modelling across all individuals from the young, middle-aged and older adult groups, respectively. We did so by contrasting the posterior distributions of model estimates derived from the Bayesian modelling approach and computing the total overlap between posterior distributions from different age groups. This was done with the *overlapping* package in *R* (Pastore, 2018). Figure S2B and Table S2 show the details of the posterior distributions by age group and model component, marking the MAP estimate and the 95% credible interval to illustrate the degree of overlap between distributions.

Table S2. Summary of model parameters obtained from Bayesian modelling across all trials from a given age group.

Task	Age Group	Measure	MAP Estimate	Lower Credible	Upper Credible	Comparison	% overlap
STM	Young	guessing	.13	.11	.15	Middle	~0
						Old	~0
	Young	SD	14.07	13.53	14.66	Middle	0
						Old	0
	Middle	guessing	.19	.17	.21	Young	~0
						Old	~0
	Middle	SD	16.43	15.89	16.99	Young	0
						Old	0
	Old	guessing	.25	.23	.27	Young	~0
						Middle	~0
	Old	SD	20.08	19.32	20.85	Young	0
						Middle	0
LTM	Young	guessing	.28	.25	.30	Middle	.38
						Old	.02
	Young	SD	15.65	14.99	16.47	Middle	0
						Old	0
	Middle	guessing	.26	.24	.28	Young	.38
						Old	~0
	Middle	SD	19.41	18.53	20.18	Young	0
						Old	~0
	Old	guessing	.33	.31	.35	Young	.02
						Middle	~0
	Old	SD	22.19	21.20	23.33	Young	0
						Middle	~0

2.3 Controlling for retrieval success

Finally, we conducted a sensitivity analysis to determine whether age effects on precision were still present when controlling for retrieval success. For the LTM task, the younger adults still had higher precision than middle-aged adults (β=.787, *95% CI* [.361, 1.213],

t(121)=3.66, *p*<.001, *d*=.67), but the older two groups did not perform significantly different from one another (*β*=-.315, *95% CI* [-.693, .063], *t*(121)=-1.648, *p*=.102, *d*=-.30). For the STM task, younger adults had significantly higher memory precision than middle-aged adults (*β*=.546, *95% CI* [.131, .960], *t*(123)=2.61, *p*=.010, *d*=.47), who in turn outperformed older adults (*β*=-.575, *95% CI* [-.929, -.222], *t*(123)=-3.22, *p*=.002, *d*=-.58).

2.4 Alternative estimation of memory precision

To further ensure that the differences in our findings for mean localisation error and κ in the LTM task did not reflect errors in the model estimates, we conducted a sensitivity analysis where an approximation of memory precision was calculated without relying on mixture modelling based on single-subject data (Cooper & Ritchey, 2019; Korkki et al., 2020b; Richter et al., 2016b). This analysis required the identification of correctly retrieved trials on the basis of mixture model components derived from modelling across all trials from all participants in our sample. Using the *CO16*_fit function from https://bayslab.com/toolbox/, we obtained the trial-by-trial probability of a given trial stemming from the uniform and the von Mises distribution, respectively. We determined which response-to-target distance corresponded to a 5% chance of belonging to the von Mises distribution and used this distance as cut-off to identify guess trials. We then took the average of the guessing cut-offs across all permutations (67° for LTM; 62° for STM) to classify each trial as forgotten or remembered. Retrieval success was then computed as the proportion of trials where the target-response distance was equal to or smaller than the derived cut-off of 67°. The standard deviation of localisation errors across all correctly remembered trials for a given participant was used as the alternative measure of imprecision, where larger values reflect poorer precision. When using this alternative memory fidelity metric, the difference between young and middle-aged participants approached significance (*β*=.377, *95% CI* [-.060, .814], *p*=.091, *d*=.31), while that between younger and older adults was highly significant (*β*=.878, *95% CI* [.443, 1.314], *p*=.22, *p*<.001, *d*=4.61). For the STM task, younger adults had greater memory precision than both middle-aged (*β*=.567, *95% CI* [.175, .958], *p*=.005, *d*=.51) and older adults (*β*=1.36, *95% CI* [.968, 1.747], *p*=.22, *p*<.001, *d*=7.96). These findings suggest that a model-informed measure of precision is still remains more sensitive to subtle declines in memory precision compared to analogue measure of memory errors. Importantly, estimating single-subject mixture model parameters is the most

powerful method to uncover these age-related changes in memory fidelity, as shown in the results of the main manuscript.

3. Exploratory Analyses Post-Review

3.1 Effects of target-lure similarity

It has previously been shown that age effects may vary as a function of target-lure similarity such that both low and particularly high degrees of feature overlap result in reduced age effects (Stark et al., 2013; Yassa et al., 2011). We therefore conducted a trial-by-trial analysis in a mixed linear model to test for such an age by similarity interaction. We classified objects into low (<2), moderate (>=2 and <3), and high (>=3) target-lure similarity bins. The model showed a main effect of age group ($F(2, 148)=12.20$, $p<.001$), with younger adults outperforming middle-aged adults ($b=.04$, $SE=.01$, $t(171)=2.77$, $p=.006$) and middle-aged adults outperforming older adults ($b=-0.06$, $SE=.01$, $t(172)=-4.57$, $p<.001$). Trial-wise Forced Choice performance was associated with target-lure similarity ($F(2, 147)=13.29$, $p<.001$). However, performance differences between age groups depended on target-lure similarity ($F(4, 18623)=3.25$ $p=.011$), suggesting that age differences are most apparent in the moderate condition. This may likely be due to a ceiling effect in the low similarity condition in the young group. Although it could be possible that the difficulty level of the high similarity condition led to younger adults declining to a level more similar to the two older groups, it is important to note that there were significantly fewer trials available for both the low and the high condition (fewer than 15 in each task) given that our task was not designed to probe the effect of target-similarity differences. As a result, we likely have insufficient statistical power to conclude that there were no longer age differences among the highest target-lure similarity trials and in fact there is a pattern of lower performance with increasing age.

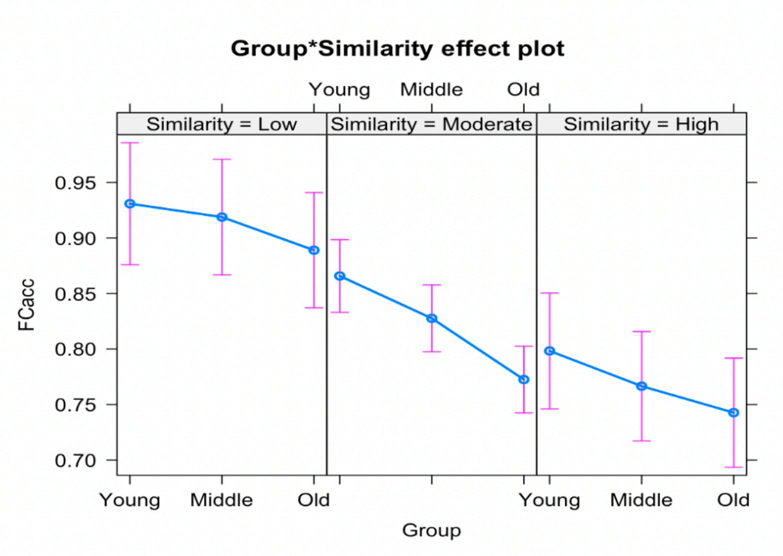

Figure S4. Estimated marginal means for the interaction effect between target-lure similarity and age group for a linear mixed model with Forced Choice mnemonic discrimination accuracy as outcome.

4. Comparison of all cognitive measures

A

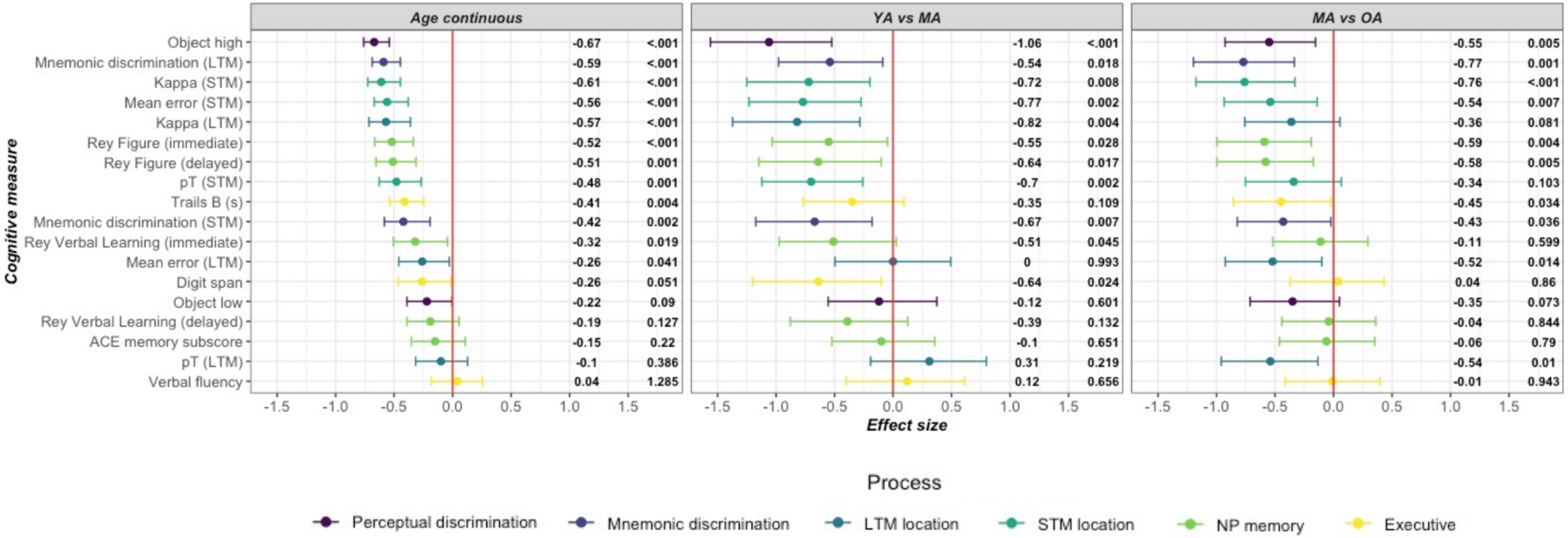

Abbreviations. LTM: long-term memory. MA: middle-aged adults. NP: neuropsychological tests. OA: older adults. pT: retrieval success. STM: short-term memory. YA: younger adults.

B

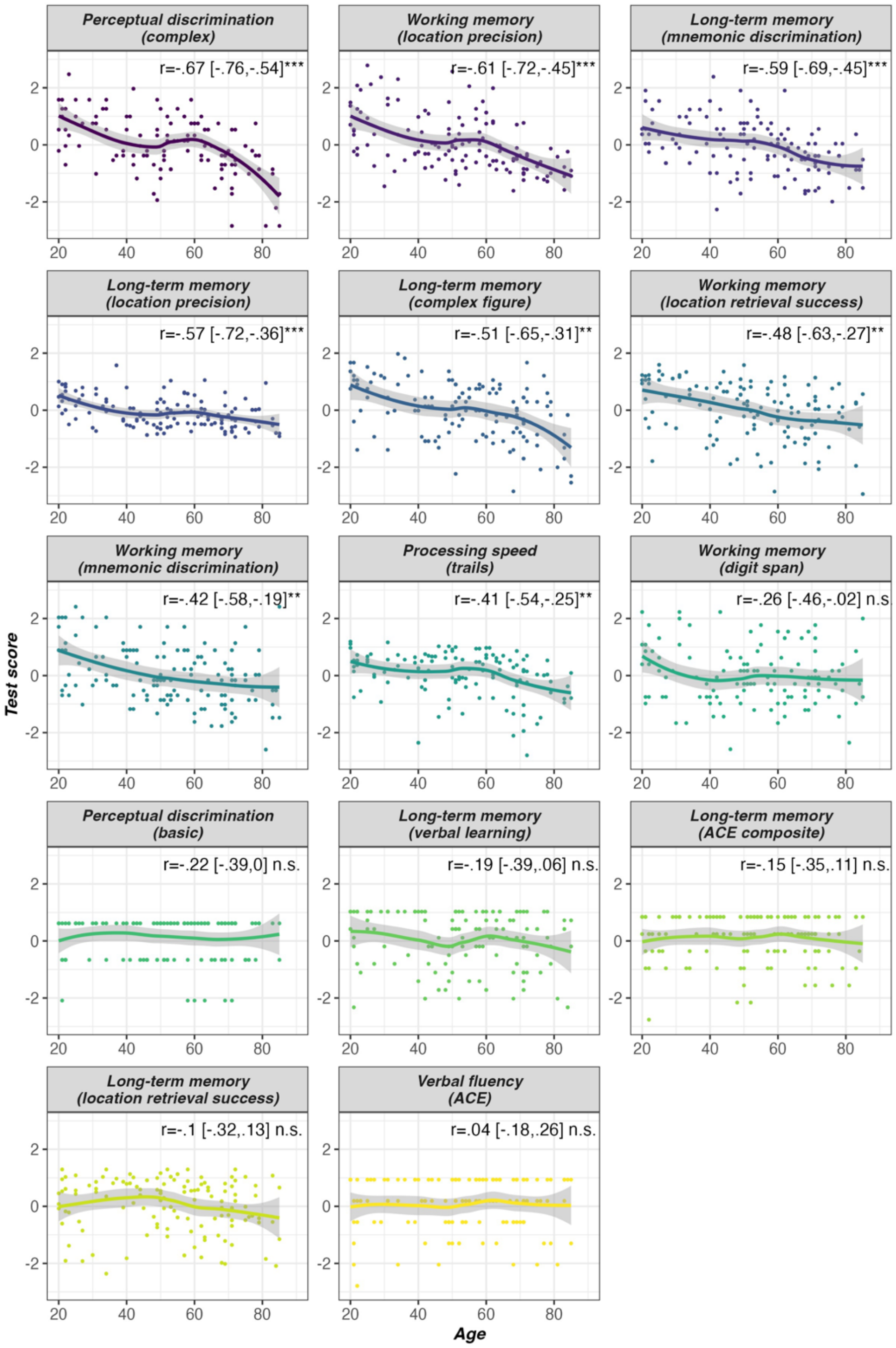

Figure S5. Summary of age effects across all cognitive measures ranked by Pearson's *r* (for the continuous age effect) and Cohen's *d* (for age group effects) obtained from a bootstrapping procedure with 10,000 samples. A. Overview of age effects with confidence intervals and coloured by cognitive domain. B. Correlation plots for the continuous age effect ordered by effect size with colours representing the size of the effect. The largest effects were found for cognitive tasks that measured perceptual and mnemonic representational quality. Effect sizes were obtained after removing extreme outliers with absolute scores larger than *z*±3. ACE: Addenbrookes Cognitive Examination.

5. Associations between precision, retrieval success and mean error

Figure S6 provides an overview of Pearson correlation coefficients between all memory measures for object-location binding. Correlations between retrieval success and precision were small to moderate. For mean absolute error, the plot suggests stronger associations between both measures of retrieval success with mean localisation error compared to the association between precision and localisation error. The differences in the strength of the association between mean localisation error and the two mixture model components demonstrated in the main manuscript (see Steiger's *z*-test: Diedenhofen & Musch, 2015; Steiger, 1980) may explain why only the κ measure could uncover reduced memory performance in middle-aged compared to younger adults in the LTM task.

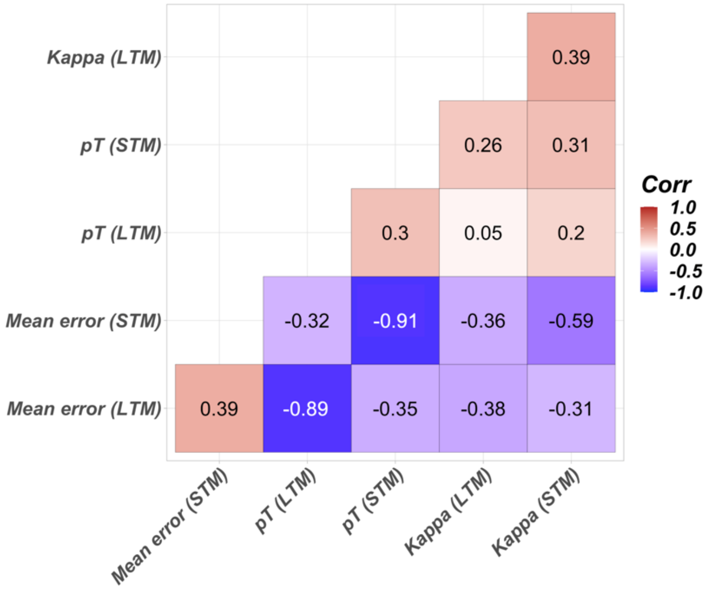

Figure S6. Correlations between different performance measures for object-location binding.

6. Details of Model Selection for Individual Differences in Memory Fidelity

Table S3. Regression models for short-term mnemonic discrimination.

	Mnemonic discrimination (STM)			Mnemonic discrimination (STM)			Mnemonic discrimination (STM)			Mnemonic discrimination (STM)		
Predictors	*Estimates*	*CI*	*p*	*Estimates*	*CI*	*p*	*Estimates*	*CI*	*p*	*Estimates*	*CI*	*p*
(Intercept)	0.00	-0.17 – 0.17	1.000	-0.00	-0.16 – 0.16	1.000	0.00	-0.16 – 0.16	1.000	-0.00	-0.16 – 0.16	1.000
Age	-0.27	-0.46 – -0.07	**0.008**	-0.21	-0.40 – -0.02	**0.031**	-0.25	-0.44 – -0.06	**0.012**	-0.20	-0.39 – -0.01	**0.036**
Education	0.06	-0.12 – 0.23	0.507	-0.01	-0.18 – 0.16	0.911	0.03	-0.14 – 0.20	0.711	-0.02	-0.19 – 0.15	0.791
Perceptual discrimination	0.13	-0.07 – 0.33	0.189	0.02	-0.18 – 0.23	0.813	0.12	-0.07 – 0.31	0.218	0.03	-0.17 – 0.23	0.774
Memory				0.33	0.14 – 0.52	**0.001**				0.28	0.09 – 0.48	**0.004**
Executive							0.25	0.08 – 0.42	**0.004**	0.21	0.04 – 0.37	**0.015**
Observations	122			122			122			122		
R^2 / R^2 adjusted	0.134 / 0.112			0.212 / 0.185			0.195 / 0.167			0.251 / 0.219		
AIC	337.641			328.221			330.787			323.940		

Table S4. Regression models for long-term mnemonic discrimination.

	Mnemonic discrimination (LTM)			Mnemonic discrimination (LTM)			Mnemonic discrimination (LTM)			Mnemonic discrimination (LTM)			Mnemonic discrimination (LTM)			Mnemonic discrimination (LTM)		
Predictors	*Estimates*	*CI*	*p*	*Estimates*	*CI*	*p*	*Estimates*	*CI*	*p*	*Estimates*	*CI*	*p*	*Estimates*	*CI*	*p*	*Estimates*	*CI*	*p*
(Intercept)	-0.00	-0.15 – 0.15	1.000	0.00	-0.15 – 0.15	1.000	-0.00	-0.14 – 0.14	1.000	-0.00	-0.14 – 0.14	1.000	-0.00	-0.14 – 0.14	1.000	-0.00	-0.14 – 0.14	1.000
Age	-0.37	-0.54 – -0.19	**<0.001**	-0.44	-0.60 – -0.29	**<0.001**	-0.33	-0.48 – -0.18	**<0.001**	-0.30	-0.47 – -0.14	**<0.001**	-0.30	-0.47 – -0.14	**<0.001**	-0.29	-0.44 – -0.14	**<0.001**
Education	0.23	0.07 – 0.38	**0.004**	0.25	0.09 – 0.40	**0.002**	0.16	0.01 – 0.31	**0.031**	0.15	0.01 – 0.30	**0.041**	0.15	0.00 – 0.30	**0.047**	0.16	0.02 – 0.31	**0.027**
Perceptual discrimination	0.19	0.01 – 0.37	**0.036**							0.07	-0.10 – 0.25	0.404	0.07	-0.10 – 0.25	0.397			
Executive				0.12	-0.04 – 0.27	0.134							0.06	-0.09 – 0.20	0.431			
Memory							0.37	0.21 – 0.53	**<0.001**	0.35	0.18 – 0.51	**<0.001**	0.34	0.17 – 0.50	**<0.001**	0.30	0.14 – 0.47	**<0.001**
Mnemonic discrimination (STM)																0.20	0.04 – 0.35	**0.013**
Observations	122			122			122			122			122			122		
R^2 / R^2 adjusted	0.324 / 0.307			0.312 / 0.294			0.408 / 0.393			0.411 / 0.391			0.415 / 0.389			0.438 / 0.419		
AIC	307.376			309.616			291.291			292.562			293.909			286.841		

Table S5. Regression models for short-term memory retrieval success.

	Retrieval success (STM)			Retrieval success (STM)			Retrieval success (STM)			Retrieval success (STM)			Retrieval success (STM)			Retrieval success (STM)		
Predictors	*Estimates*	*CI*	*p*	*Estimates*	*CI*	*p*	*Estimates*	*CI*	*p*	*Estimates*	*CI*	*p*	*Estimates*	*CI*	*p*	*Estimates*	*CI*	*p*
(Intercept)	-0.00	-0.17 – 0.17	1.000	-0.00	-0.16 – 0.16	1.000	-0.00	-0.17 – 0.17	1.000	-0.00	-0.16 – 0.16	1.000	-0.00	-0.16 – 0.16	1.000	-0.00	-0.16 – 0.16	0.976
Age	-0.30	-0.50 – -0.11	**0.002**	-0.33	-0.49 – -0.17	**<0.001**	-0.31	-0.49 – -0.13	**0.001**	-0.29	-0.46 – -0.12	**0.001**	-0.26	-0.45 – -0.08	**0.006**	-0.24	-0.43 – -0.05	**0.013**
Education	0.01	-0.16 – 0.18	0.931	-0.01	-0.17 – 0.15	0.891	-0.01	-0.18 – 0.16	0.910	-0.03	-0.20 – 0.13	0.703	-0.04	-0.20 – 0.13	0.647	-0.03	-0.19 – 0.13	0.730
Perceptual discrimination	0.13	-0.07 – 0.32	0.197										0.08	-0.12 – 0.27	0.438	0.09	-0.09 – 0.28	0.335
Executive				0.32	0.16 – 0.48	**<0.001**				0.30	0.13 – 0.47	**<0.001**	0.30	0.13 – 0.46	**0.001**	0.27	0.11 – 0.44	**0.002**
Memory							0.18	-0.01 – 0.36	0.060	0.11	-0.07 – 0.29	0.214	0.09	-0.10 – 0.28	0.335			
Mnemonic discrimination (STM)																0.16	-0.02 – 0.33	0.083
Observations	121			121			121			121			121			120		
R^2 / R^2 adjusted	0.147 / 0.125			0.234 / 0.215			0.161 / 0.139			0.245 / 0.219			0.249 / 0.216			0.263 / 0.231		
AIC	333.108			320.049			331.168			320.434			321.799			317.916		

Table S6. Regression models for long-term memory retrieval success.

	Retrieval success (LTM)			Retrieval success (LTM)			Retrieval success (LTM)			Retrieval success (LTM)			Retrieval success (LTM)			Retrieval success (LTM)		
Predictors	*Estimates*	*CI*	*p*	*Estimates*	*CI*	*p*	*Estimates*	*CI*	*p*	*Estimates*	*CI*	*p*	*Estimates*	*CI*	*p*	*Estimates*	*CI*	*p*
(Intercept)	0.00	-0.17 – 0.17	1.000	0.00	-0.18 – 0.18	1.000	0.00	-0.17 – 0.17	1.000	0.00	-0.16 – 0.16	1.000	0.00	-0.16 – 0.16	1.000	0.00	-0.15 – 0.15	1.000
Age	-0.02	-0.22 – 0.18	0.826	-0.14	-0.32 – 0.04	0.119	-0.01	-0.19 – 0.17	0.931	0.05	-0.14 – 0.25	0.590	0.06	-0.14 – 0.25	0.572	0.24	0.05 – 0.42	**0.013**
Education	0.06	-0.12 – 0.24	0.504	0.09	-0.09 – 0.27	0.324	0.01	-0.16 – 0.18	0.906	-0.01	-0.18 – 0.17	0.951	-0.01	-0.18 – 0.16	0.906	-0.08	-0.23 – 0.08	0.326
Perceptual discrimination	0.28	0.07 – 0.49	**0.008**							0.16	-0.04 – 0.36	0.124	0.16	-0.04 – 0.37	0.121	0.12	-0.06 – 0.31	0.184
Executive				0.14	-0.04 – 0.32	0.135							0.07	-0.10 – 0.24	0.425			
Memory							0.42	0.23 – 0.61	**<0.001**	0.37	0.18 – 0.57	**<0.001**	0.36	0.16 – 0.56	**<0.001**	0.21	0.02 – 0.39	**0.027**
Mnemonic discrimination (LTM)																0.54	0.35 – 0.72	**<0.001**
Observations	120			120			120			120			120			120		
R^2 / R^2 adjusted	0.096 / 0.073			0.058 / 0.034			0.182 / 0.161			0.199 / 0.171			0.203 / 0.168			0.371 / 0.343		
AIC	337.376			342.318			325.461			324.978			326.303			297.995		

Table S7. Regression models for short-term memory precision.

	Precision (STM)			Precision (STM)			Precision (STM)			Precision (STM)			Precision (STM)			Precision (STM)			Precision (STM)		
Predictors	*Estimates*	*CI*	*p*	*Estimates*	*CI*	*p*	*Estimates*	*CI*	*p*	*Estimates*	*CI*	*p*	*Estimates*	*CI*	*p*	*Estimates*	*CI*	*p*	*Estimates*	*CI*	*p*
(Intercept)	0.00	-0.15 – 0.15	1.000	0.00	-0.14 – 0.14	1.000	0.00	-0.15 – 0.15	1.000	0.00	-0.14 – 0.14	1.000	0.00	-0.14 – 0.14	1.000	0.00	-0.14 – 0.14	1.000	0.01	-0.13 – 0.15	0.891
Age	-0.40	-0.57 – -0.22	**<0.001**	-0.45	-0.60 – -0.31	**<0.001**	-0.41	-0.57 – -0.25	**<0.001**	-0.40	-0.55 – -0.24	**<0.001**	-0.35	-0.52 – -0.18	**<0.001**	-0.40	-0.56 – -0.24	**<0.001**	-0.39	-0.54 – -0.23	**<0.001**
Education	0.19	0.03 – 0.34	**0.018**	0.18	0.04 – 0.33	**0.015**	0.16	0.00 – 0.31	**0.049**	0.14	-0.01 – 0.29	0.066	0.13	-0.02 – 0.28	0.090	0.14	-0.01 – 0.29	0.068	0.14	-0.01 – 0.29	0.059
Perceptual discrimination	0.18	0.01 – 0.36	**0.039**										0.12	-0.05 – 0.29	0.171						
Executive				0.27	0.12 – 0.41	**<0.001**				0.24	0.09 – 0.38	**0.002**	0.23	0.09 – 0.38	**0.002**	0.23	0.08 – 0.39	**0.004**	0.22	0.07 – 0.37	**0.005**
Memory							0.23	0.06 – 0.39	**0.007**	0.18	0.02 – 0.34	**0.030**	0.14	-0.02 – 0.31	0.091	0.18	0.02 – 0.34	**0.032**	0.18	0.01 – 0.35	**0.039**
Retrieval success (STM)																0.00	-0.16 – 0.17	0.966			
Mnemonic discrimination (STM)																			0.04	-0.12 – 0.20	0.632
Observations	122			122			122			122			122			122			121		
R^2 / R^2 adjusted	0.323 / 0.306			0.367 / 0.351			0.340 / 0.324			0.392 / 0.371			0.402 / 0.376			0.392 / 0.366			0.399 / 0.373		
AIC	307.621			299.409			304.470			296.506			296.524			298.504			294.556		

Table S8. Regression models for long-term memory precision.

	Precision (LTM)			Precision (LTM)			Precision (LTM)			Precision (LTM)			Precision (LTM)			Precision (LTM)			Precision (LTM)			Precision (LTM)		
Predictors	*Estimates*	*CI*	*p*	*Estimates*	*CI*	*p*	*Estimates*	*CI*	*p*	*Estimates*	*CI*	*p*	*Estimates*	*CI*	*p*	*Estimates*	*CI*	*p*	*Estimates*	*CI*	*p*	*Estimates*	*CI*	*p*
(Intercept)	-0.00	-0.16 – 0.16	1.000	-0.00	-0.16 – 0.16	1.000	-0.00	-0.14 – 0.14	1.000	-0.00	-0.15 – 0.15	1.000	-0.00	-0.14 – 0.14	1.000	-0.00	-0.14 – 0.14	1.000	0.00	-0.14 – 0.14	1.000	0.00	-0.14 – 0.14	1.000
Age	-0.41	-0.59 – -0.22	**<0.001**	-0.47	-0.63 – -0.31	**<0.001**	-0.32	-0.48 – -0.16	**<0.001**	-0.32	-0.49 – -0.15	**<0.001**	-0.32	-0.48 – -0.16	**<0.001**	-0.25	-0.42 – -0.08	**0.005**	-0.24	-0.41 – -0.08	**0.005**	-0.19	-0.37 – -0.01	**0.041**
Education	0.04	-0.13 – 0.20	0.672	0.05	-0.11 – 0.22	0.518	-0.04	-0.19 – 0.11	0.592	-0.04	-0.19 – 0.12	0.625	-0.04	-0.19 – 0.11	0.599	-0.07	-0.22 – 0.08	0.377	-0.07	-0.22 – 0.08	0.358	-0.09	-0.24 – 0.06	0.230
Perceptual discrimination	0.14	-0.05 – 0.32	0.153							-0.00	-0.18 – 0.18	0.987												
Executive				0.03	-0.13 – 0.20	0.678				-0.04	-0.19 – 0.11	0.583												
Memory							0.43	0.27 – 0.59	**<0.001**	0.44	0.26 – 0.61	**<0.001**	0.46	0.28 – 0.64	**<0.001**	0.39	0.22 – 0.55	**<0.001**	0.36	0.19 – 0.53	**<0.001**	0.33	0.16 – 0.50	**<0.001**
Retrieval success (LTM)													-0.07	-0.23 – 0.09	0.367									
Precision (STM)																0.17	-0.01 – 0.35	0.062				0.15	-0.02 – 0.33	0.091
Mnemonic discrimination (LTM)																			0.21	0.02 – 0.39	**0.029**	0.19	0.01 – 0.37	**0.041**
Observations	120			120			120			120			120			120			120			120		
R^2 / R^2 adjusted	0.246 / 0.227			0.234 / 0.214			0.380 / 0.364			0.382 / 0.355			0.385 / 0.363			0.399 / 0.378			0.406 / 0.385			0.421 / 0.395		
AIC	315.623			317.568			292.081			295.762			293.227			290.431			289.056			288.031		

7. References

Bays, P. M., Catalao, R. F. G., & Husain, M. (2009). The precision of visual working memory is set by allocation of a shared resource. *Journal of Vision*, *9(10)*, 1–11. https://doi.org/10.1167/9.10.7.

Cooper, R. A., & Ritchey, M. (2019). Cortico-hippocampal network connections support the multidimensional quality of episodic memory. *ELife*, *8*, 1–37. https://doi.org/10.7554/elife.45591

Diedenhofen, B., & Musch, J. (2015). cocor: A Comprehensive Solution for the Statistical Comparison of Correlations. *PLoS ONE*, *10*(4). https://doi.org/10.1371/journal.pone.0121945

Gellersen, H. M., Coughlan, G., Hornberger, M., & Simons, J. S. (2021). Memory precision of object-location binding is unimpaired in APOE ε4-carriers with spatial navigation deficits. *Brain Communications*, *3*(2), fcab087. https://doi.org/10.1101/2020.12.18.423245

Harlow, I. M., & Donaldson, D. I. (2013). Source accuracy data reveal the thresholded nature of human episodic memory. *Psychological Bulletin Review*, *20*, 318–325. https://doi.org/10.3758/s13423-012-0340-9

Korkki, S. M., Richter, F. R., Jeyarathnarajah, P., & Simons, J. S. (2020a). Healthy Ageing Reduces the Precision of Episodic Memory Retrieval. *Psychology and Aging*, *35*(1), 124–142. https://doi.org/10.1037/pag0000432

Pastore, M. (2018). Overlapping: a R package for estimating overlapping in empirical distributions. *Journal of Open Source Software*, *3*(32), 1023. https://doi.org/10.21105/joss.01023

Peich, M. C., Husain, M., & Bays, P. M. (2013). Age-related decline of precision and binding in visual working memory. *Psychology and Aging*, *28*(3), 729–743. https://doi.org/10.1037/a0033236

Pertzov, Y., Heider, M., Liang, Y., & Husain, M. (2015). Effects of healthy ageing on precision and binding of object location in visual short term memory. *Psychology and Aging*, *30*(1), 26–35. https://doi.org/10.1037/a0038396

Richter, F. R., Cooper, R. A., Bays, P. M., & Simons, J. S. (2016). *Distinct neural mechanisms underlie the success , precision , and vividness of episodic memory*. 1–18. https://doi.org/10.7554/eLife.18260

Stark, S. M., Yassa, M. a., Lacy, J. W., & Stark, C. E. L. (2013). A task to assess behavioral pattern separation (BPS) in humans. *Neuropsychologia*, *51*(12), 2442–2449. https://doi.org/10.1016/j.neuropsychologia.2012.12.014.A

Steiger, J. H. (1980). Tests for comparing elements of a correlation matrix. *Psychological Bulletin*, *87*, 245–251.

Yassa, M. A., Lacy, J. W., Stark, S. M., Albert, M. S., Gallagher, M., & Stark, C. E. (2011). Pattern separation deficits associated with increased hippocampal CA3 and dentate gyrus activity in nondemented older adults. *Hippocampus*, *21*(9), 968-979.

Zhang, W., & Luck, S. J. (2008). *Discrete fixed-resolution representations in visual working memory*. *453*(May), 233–236. https://doi.org/10.1038/nature06860
